# Characterization of smoke and dust episode over West Africa: comparison of MERRA-2 modeling with multiwavelength Mie–Raman lidar observations

**DOI:** 10.5194/amt-11-949-2018

**Published:** 2018-02-16

**Authors:** Igor Veselovskii, Philippe Goloub, Thierry Podvin, Didier Tanre, Arlindo da Silva, Peter Colarco, Patricia Castellanos, Mikhail Korenskiy, Qiaoyun Hu, David N. Whiteman, Daniel Pérez-Ramírez, Patrick Augustin, Marc Fourmentin, Alexei Kolgotin

**Affiliations:** 1 Physics Instrumentation Center of GPI, Troitsk, Moscow, Russia; 2 Joint Center for Earth Systems Technology, UMBC, Baltimore, USA; 3 NASA Goddard Space Flight Center, Greenbelt, USA; 4 Laboratoire d’Optique Atmosphérie, Université de Lille-CNRS, Villeneuve d’Ascq, France; 5 Universities Space Research Association, Columbia, Maryland, USA; 6 Applied Physics Department, University of Granada, Spain; 7 Laboratoire de Physico-Chimie de l’Atmosphère, Université du Littoral Côte d’Opale, France

## Abstract

Observations of multiwavelength Mie–Raman lidar taken during the SHADOW field campaign are used to analyze a smoke–dust episode over West Africa on 24–27 December 2015. For the case considered, the dust layer extended from the ground up to approximately 2000 m while the elevated smoke layer occurred in the 2500–4000 m range. The profiles of lidar measured backscattering, extinction coefficients, and depolarization ratios are compared with the vertical distribution of aerosol parameters provided by the Modern-Era Retrospective Analysis for Research and Applications, version 2 (MERRA-2). The MERRA-2 model simulated the correct location of the near-surface dust and elevated smoke layers. The values of modeled and observed aerosol extinction coefficients at both 355 and 532 nm are also rather close. In particular, for the episode reported, the mean value of difference between the measured and modeled extinction coefficients at 355 nm is 0.01 km^−1^ with SD of 0.042 km^−1^. The model predicts significant concentration of dust particles inside the elevated smoke layer, which is supported by an increased depolarization ratio of 15 % observed in the center of this layer. The modeled at 355 nm the lidar ratio of 65 sr in the near-surface dust layer is close to the observed value (70 ± 10) sr. At 532 nm, however, the simulated lidar ratio (about 40 sr) is lower than measurements (55 ± 8 sr). The results presented demonstrate that the lidar and model data are complimentary and the synergy of observations and models is a key to improve the aerosols characterization.

## 1 Introduction

Atmospheric aerosols are an important factor influencing the Earth’s radiative budget, though its impact is still highly uncertain due largely to the complicated mechanisms of aerosol–cloud interaction. Aerosol particles serve as cloud condensation nuclei and ice-nucleating particles, creating a strong impact on cloud and precipitation formation. However, different aerosol types differ significantly in their ability to initiate drop and ice crystal nucleation. There is thus a clear need for a better knowledge on vertically resolved optical, physical, and chemical aerosol properties. Lidar is a recognized instrument for vertical profiling of aerosol properties, and the possibility to invert lidar observations at several wavelengths to aerosol microphysical properties has been extensively studied both theoretically and experimentally over the past two decades (e.g., Müller et al., 1999, 2016; Veselovskii et al., 2002; Böckman et al., 2005). These studies revealed the importance of using Raman or HSRL (high spectral resolution lidar) systems, which allow independent measurements of aerosol extinction and backscattering coefficients to be made. At present, the most practical configuration of Raman (HSRL) lidar is based on a triple wavelength Nd:YAG laser. Such a lidar provides the so-called 3*β* + 2*α* set of observations, including three backscattering (355, 532, 1064 nm) and two extinction (355, 532 nm) coefficients.

However, the problem of inversion of 3*β* + 2*α* observations is underdetermined (Chemyakin et al., 2016; Alexandrov and Mishchenko, 2017). As a result, instead of a unique solution, a family of solutions should be considered, leading to an increase in retrieval uncertainties. Still the estimation of volume density (*V*) and effective radius (*r*_eff_) with uncertainty below 30 % is possible, especially when the fine mode in the particle size distribution (PSD) is predominant (e.g., Veselovskii et al., 2004; Müller et al., 2005, 2016; Pérez-Ramírez et al., 2013). The refractive index (RI) can be also estimated from the measurements, although the uncertainty of such estimation is significant: for the real part (*m*_R_) of RI the uncertainty is normally about ± 0.05 and for the imaginary part (*m*_I_) it is about 50 % when *m*_I_ > 0.01 (Veselovskii et al., 2004; Müller et al., 2016). Proposed improvements of inversion schemes were considered in recent publications (Chemyakin et al., 2014; Kolgotin et al., 2016), but these improvements are not able to resolve the fundamental issue: the information content of 3*β* + 2*α* observations is insufficient to support exact solution of the problem and additional information should be used in retrievals to improve the accuracy of the retrieved products (Veselovskii et al., 2005; Burton et al., 2016; Alexandrov and Mishchenko, 2017; Kahnert and Andersson, 2017).

We should recall also that in the inversion schemes considered, the RI is normally assumed to be spectrally and size independent, which is generally not the case in the atmosphere. The irregularity of the particles shape can be also a significant error source. Moreover, the volume density and effective radius obtained from 3*β* + 2*α* observations are attributed to the whole size distribution, which is of limited practical use because of the importance of characterizing the particle properties separately for the fine and coarse modes. Considering these issues makes the inverse problem even more underdetermined, emphasizing the need for additional input information.

One opportunity to get this additional information is by combining the lidar observations with aerosol transport models (Kahnert and Andersson, 2017). Models provide the vertical distribution of mass mixing ratios of chemical aerosol components, which can be used as “initial guess” in the inversion scheme. Modern-Era Retrospective Analysis for Research and Applications, version 2 (MERRA-2), offers a unique opportunity to provide such an “initial guesses” of the vertical structure of aerosol chemical composition. MERRA-2 is produced with NASA’s global Earth system model, GEOS-5 (Goddard Earth Observing System version 5) (Gelaro et al., 2017) and includes an online coupling with the Goddard Chemistry, Aerosol, Radiation and Transport model (GOCART), which allows for assimilation of aerosol optical depth (AOD) from spaceborne and surface instruments such as MODIS, AVHRR, MISR, and AERONET (Randles et al., 2017). The fundamental data that MERRA-2 provides are vertical profiles of the mass mixing ratios of five aerosol components: dust, sea salt (SS), black and organic carbon (BC and OC), and sulfate (SU) aerosols. The main optical parameters related to lidar measurements, such as aerosol extinction and backscattering coefficients can be calculated basing on these data. The principal question arising, however, is how well the reanalysis reproduces independent observations and thus can provide a realistic initial guess for a lidar inversion scheme. Buchard et al. (2017) and Randles et al. (2017) extensively validated MERRA-2 with independent surface and aircraft observations of particulate matter (PM_2.5_) and AOD, as well as space-based observations of absorption aerosol optical depth and aerosol index. The extinction profiles derived from airborne HSRL measurements were also compared with modeling, finding generally good agreement between the observations and MERRA-2.

For global validation of the aerosol vertical distribution, the modeled profiles of attenuated backscatter were compared to spaceborne Cloud–Aerosol Lidar with Orthogonal Polarization (CALIOP) observations (Winker et al., 2009), and a good consistency between simulations and observations was reported (Nowottnick et al., 2015; Buchard et al., 2017). Additional opportunities for model validation are provided by ground-based multiwavelength Raman or HSRL systems. Such lidars by their nature have limited spatial coverage but are well suited for the characterization of the vertical distribution of particle properties at a chosen location.

In our paper, we consider Raman lidar observations taken during a smoke–dust episode over West Africa in December 2015 during the SHADOW (SaHAran Dust Over West Africa) campaign (Veselovskii et al., 2016) and compare the vertical profiles of particle parameters with MERRA-2. The simultaneous presence of dust and smoke layers in the atmosphere provides an opportunity to test the ability of the model to reproduce the vertical structure of aerosol properties over the observation site.

## 2 Measurement setup and data analysis

### 2.1 Observation site

The observation site is located at the Institute for Research and Development Center (IRDC), Mbour, Senegal (14° N, 17° W). Information about the SHADOW campaign and instruments at the IRDC site can be found in the recent publication by Veselovskii et al. (2016). During the SHADOW campaign data from three lidar instruments were available:

– Cimel CE-370 micropulse lidar (www.cimel.fr) operated 24 h day^−1^ at 532 nm, allowing real-time monitoring of aerosol and cloud layers.
– Doppler lidar Windcube WLS 100 (www.leosphere.com) provided continuous monitoring of the wind field in the range from 100 m to 5 km with 50 m range resolution at 1543 nm wavelength.
– Multiwavelength Mie–Raman polarization lidar LILAS (LIlle Lidar AtmosphereS) allowed simultaneous detection of elastic and Raman backscatter signals and thus provided 3*β* + 2*α* observations along with depolarization ratio at 532 nm.

LILAS measurements were performed from inside a laboratory building through a window at an angle of 47° with respect to the horizon. Acquiring Raman backscatter at 408 nm also permits profiling of the water vapor mixing ratio (WVMR) (Whiteman et al., 1992). For calibration of the water vapor channel, radiosonde launches from Dakar (about 70 km away from Mbour) were used. The large separation between the lidar and radiosonde locations prevented an accurate calibration, so the WVMR data were used mainly to monitor the relative change of the water vapor content. The temporal resolution of the measurements was approximately 3 min. The backscattering coefficients and depolarization ratio were calculated with range resolution 7.5 m (corresponding to a vertical spatial resolution of 5.5. m). The height resolution of the extinction coefficient measurements varied with height from 50 m (at 1000 m) to 125 m (at 7000 m). The measurements were performed mainly in the nighttime. In the daytime, the Raman measurements at 532 nm were possible only up to 2–3 km height, so continuous night- and daytime Raman measurements were performed only for selected episodes.

The particle extinction (*α*) and backscattering (*β*) coefficients at 355 and 532 nm are calculated from elastic and Raman backscatter signals, as described in Ansmann et al. (1992). Backscattering coefficients at 1064 nm (*β*_1064_) were calculated by the Klett method (Klett, 1981).

In the data analysis both volume (*δ*^v^) and particle (*δ*) depolarization ratios are considered. These ratios are defined as

(1) $$\delta^{\text{v}}=\frac{\beta_{⊥}^{\text{p}}+\beta_{⊥}^{\text{m}}}{\beta_{\text{II}}^{\text{p}}+\beta_{\text{II}}^{\text{m}}}=C\frac{P_{⊥}}{P_{\text{II}}},$$

(2) $$\delta =\frac{\beta_{⊥}^{\text{p}}}{\beta_{\text{II}}^{\text{p}}}.$$

Here *P* is the power of the elastic backscatter signal. Superscripts “p” and “m” indicate particle and molecule backscattering, while subscripts “⊥” and “II” indicate cross- and copolarized components, and *C* is the calibration constant. Particle depolarization is calculated as suggested by Freudenthaler et al. (2009):

(3) $$\delta =\frac{\left(1+\delta^{\text{m}}\right)\delta^{\text{v}}R-\left(1+\delta^{\text{v}}\right)\delta^{\text{m}}}{\left(1+\delta^{\text{m}}\right)R-\left(1+\delta^{\text{v}}\right)}.$$

Here *δ*^m^ is the molecular depolarization ratio and *R* is the aerosol scattering ratio:

(4) $$R=\frac{\beta^{\text{p}}+\beta^{\text{m}}}{\beta^{\text{m}}}.$$

For further convenience we will use the notations $\beta =\beta_{\text{II}}^{\text{p}}+\beta_{⊥}^{\text{p}}$ and $\alpha =\alpha^{\text{p}}$. To characterize the spectral dependence of *β* and *α*, the backscattering and extinction Ångström exponents (BAE and EAE) for wavelengths λ_1_ and λ_2_ are calculated as

(5) $$A^{\beta }=\frac{\ln\left(\frac{\beta_{\lambda_{1}}}{\beta_{\lambda_{2}}}\right)}{\ln\left(\frac{\lambda_{2}}{\lambda_{1}}\right)},\;A^{\alpha }=\frac{\ln\left(\frac{\alpha_{\lambda_{1}}}{\alpha_{\lambda_{2}}}\right)}{\ln\left(\frac{\lambda_{2}}{\lambda_{1}}\right)}.$$

The lidar-derived backscattering and extinction coefficients can be inverted to the particle microphysical properties, as described at Veselovskii et al. (2002). The only constraints on the permitted RI and the PSD are that the RI is considered to be wavelength independent and that the concentration of the particles with radii below some *r*_min_ and above some *r*_max_ is zero, where the values of these radii are found in the process of inversion.

### 2.2 MERRA-2 aerosol reanalysis

The MERRA-2 simulations of aerosol properties over the observation site were made using the GOCART model (Chin et al., 2002) integrated within GEOS-5. The model includes representations of dust, SS, BC, OC, and SU aerosols. The aerosol components are assumed to be externally mixed. The optical properties of these aerosol components are summarized in Appendix A. Sulfate and carbonaceous aerosols are both assumed to be in the fine mode. Sea salt and dust are both represented by five size bins spanning 0.1–10 μm radius for dust and 0.03–10 μm dry radius for sea salt, allowing for the simulation of both the fine and coarse fractions of each. A more complete description of how GOCART is implemented in GEOS-5 is provided in Colarco et al. (2010), which also includes a detailed evaluation of the model with respect to MODIS, MISR, and AERONET aerosol optical depth observations.

The aerosol optical properties are primarily based on Mie calculations using the particle properties as in Colarco et al. (2010) and Chin et al. (2002), with spectral refractive indices from the Optical Properties of Aerosols and Clouds (OPAC; Hess et al., 1998) database. However, for dust, non-spherical optical properties derived from an offline database are used (Colarco et al., 2014). For SS, SU, and the hydrophilic portion of carbonaceous aerosol, hygroscopic growth is considered following Chin et al. (2002), with growth factors from OPAC (Gerber, 1985). The RI for organic carbon is based on the 100 % brown carbon case from Hammer et al. (2016) and it is implemented as described in Colarco et al. (2017).

The sources of aerosols in the model include wind-speed-based emissions of dust and sea salt, fossil fuel combustion, biomass burning, biofuel consumption, biogenic particulate organic matter, and oxidation of dimethyl sulfide and SO_2_, which includes volcanic sources. Aerosol sinks include convective scavenging, dry deposition, and wet removal, where aerosol hygroscopic growth is considered in the calculation of particle fall velocity and deposition velocity. The model resolution is 0.5° × 0.625° latitude by longitude with 72 hybrid-eta layers from the surface to 0.01 hPa. Additional details of the simulation can be found in Randles et al. (2017) and Buchard et al. (2017).

In MERRA-2, aerosol and meteorological observations are jointly assimilated within GEOS-5. Aerosols are assimilated by means of analysis splitting and the local displacement ensemble methodology (Buchard et al., 2015, 2016). The system assimilated MODIS, AVHRR, MISR, and AERONET 550 nm AOD. AERONET measurements are interpolated to 550 nm using the Ångström relationship and the closest available channels, generally 500 and 675 nm. The assimilation determines an AOD increment, which corrects the model AOD in a way that minimizes the differences between the model and observations. The AOD increment both corrects for misplaced aerosol plumes and scales the aerosol mass mixing ratio to match the observations. The 2-D AOD increment does not contain enough information to correct either the vertical distribution of aerosols or the aerosol composition. Thus, the model determines the aerosol speciation, optical properties, and vertical distributions, while the AOD increments modulate the aerosol mass. Thus, the assimilated aerosol distributions and physical and optical properties arise from the forecast model assumptions and the formulation of the aerosol data assimilation algorithm.

## 3 Experimental results

**Figure 1.**
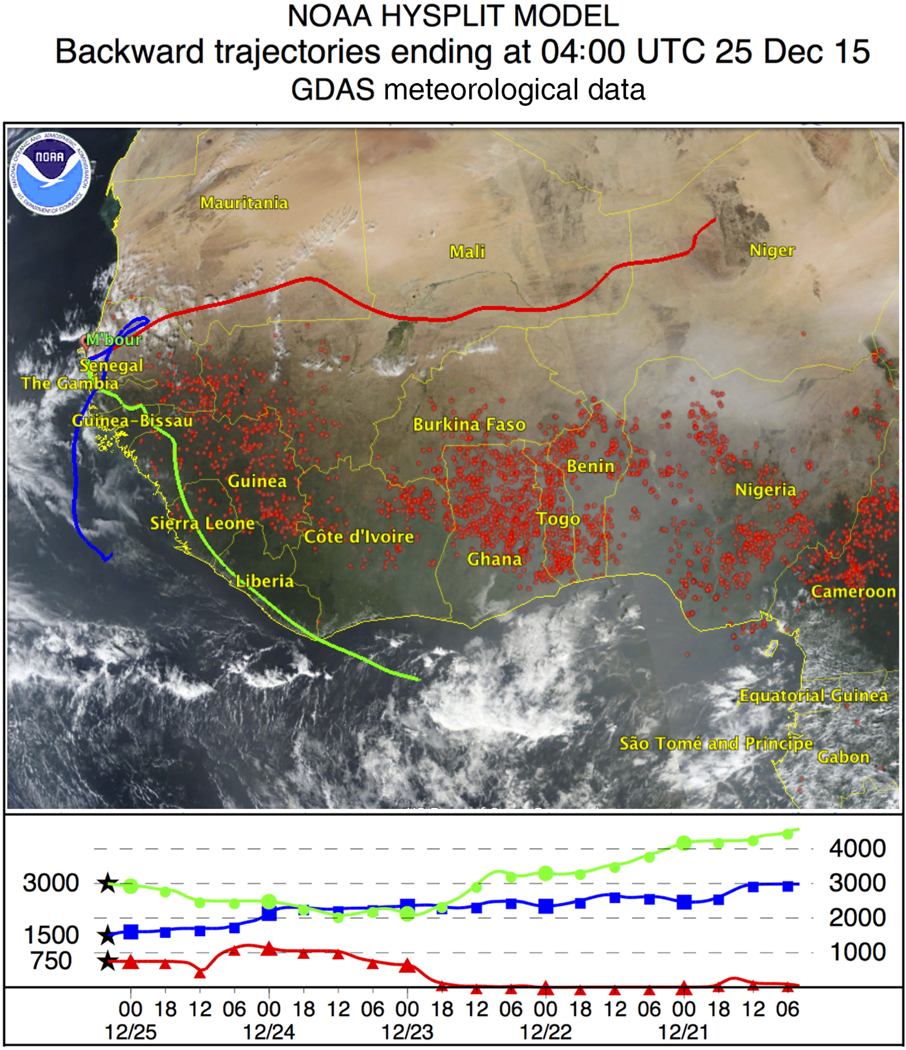
Five-day backward trajectories for the air mass in Mbour at altitudes 750, 1500, and 3500 m, on 25 December 2015 at 04:00 UTC, together with the map of forest fires on 20 December 2015.

The smoke layers from forest fires near the Equator were regularly observed over the instrumentation site during the wintertime measurement sessions made in December 2015–January 2016. In our study we will focus on a strong smoke episode that occurred on 24–27 December 2015. Air mass back trajectories over Mbour on 25 December 2015 at 04:00 UTC are shown in Fig. 1 together with map of fires on 20 December 2015 (https://worldview.earthdata.nasa.gov).

**Figure 2.**
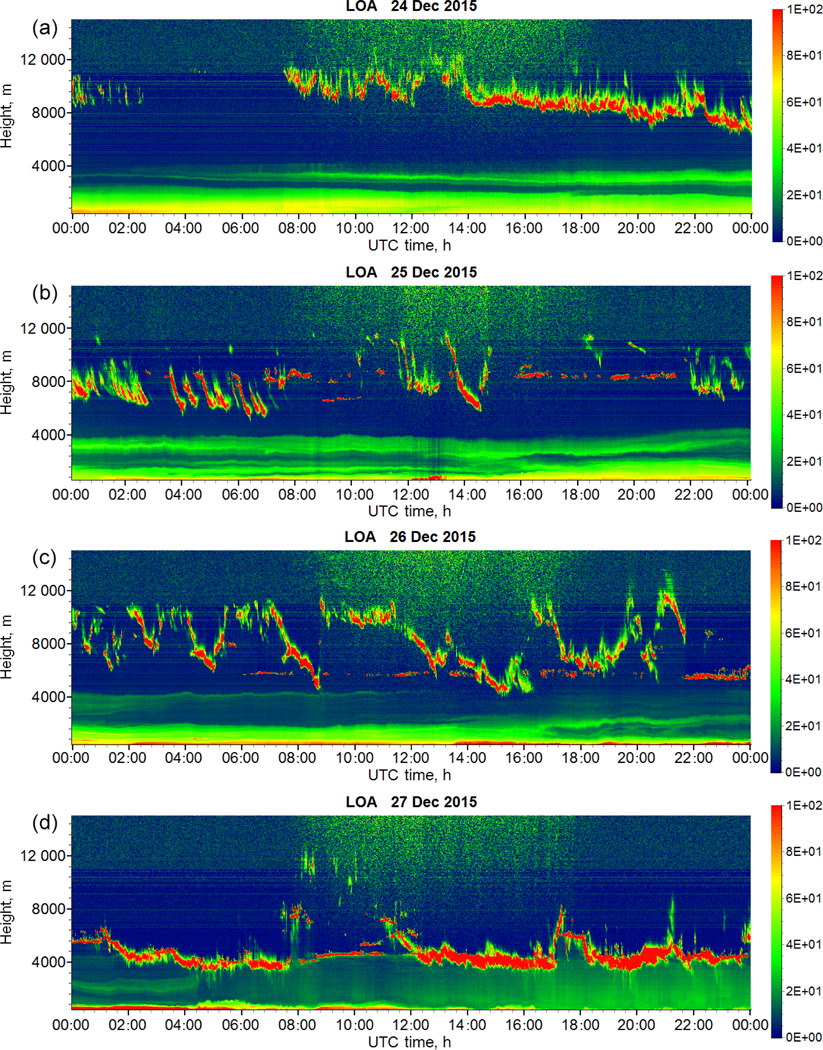
Range-corrected lidar signal (in arbitrary units) of Cimel MPL for 24–27 December 2015.

The air masses below 1000 m (red line) are transported over the desert and are strongly loaded by dust, while air masses at 3000 m (green line) arrive from the south and pass over the regions of forest fires and thus can transport smoke particles. The Cimel MPL operated continuously through the period of 24–27 December and thus monitored the arrival and evolution of the smoke layer, as shown in Fig. 2. An elevated smoke layer appears on 24 December around 00:00 UTC. The aerosol layer becomes thicker during the day but remains confined to the height interval of 2.5–4.0 km and stays well separated from the dust layer, which extends from the ground to approximately 2.0 km. This structure of the layers is preserved throughout 25 December, as well. Cirrus clouds appear at 08:00 UTC on 24 December at a height of 8 km, soon after the smoke layer arrival (Fig. 2a) and persist throughout the smoke episode. After 12:00 UTC on 24 December the clouds start descending and by 07:00 UTC on 25 December the cloud base is below 6 km (Fig. 2b). On 26 December strong precipitation of ice particles occurs (Fig. 2c) and, finally, on 27 December the cloud is located at the top of the smoke–dust layer (Fig. 2d).

**Figure 3.**
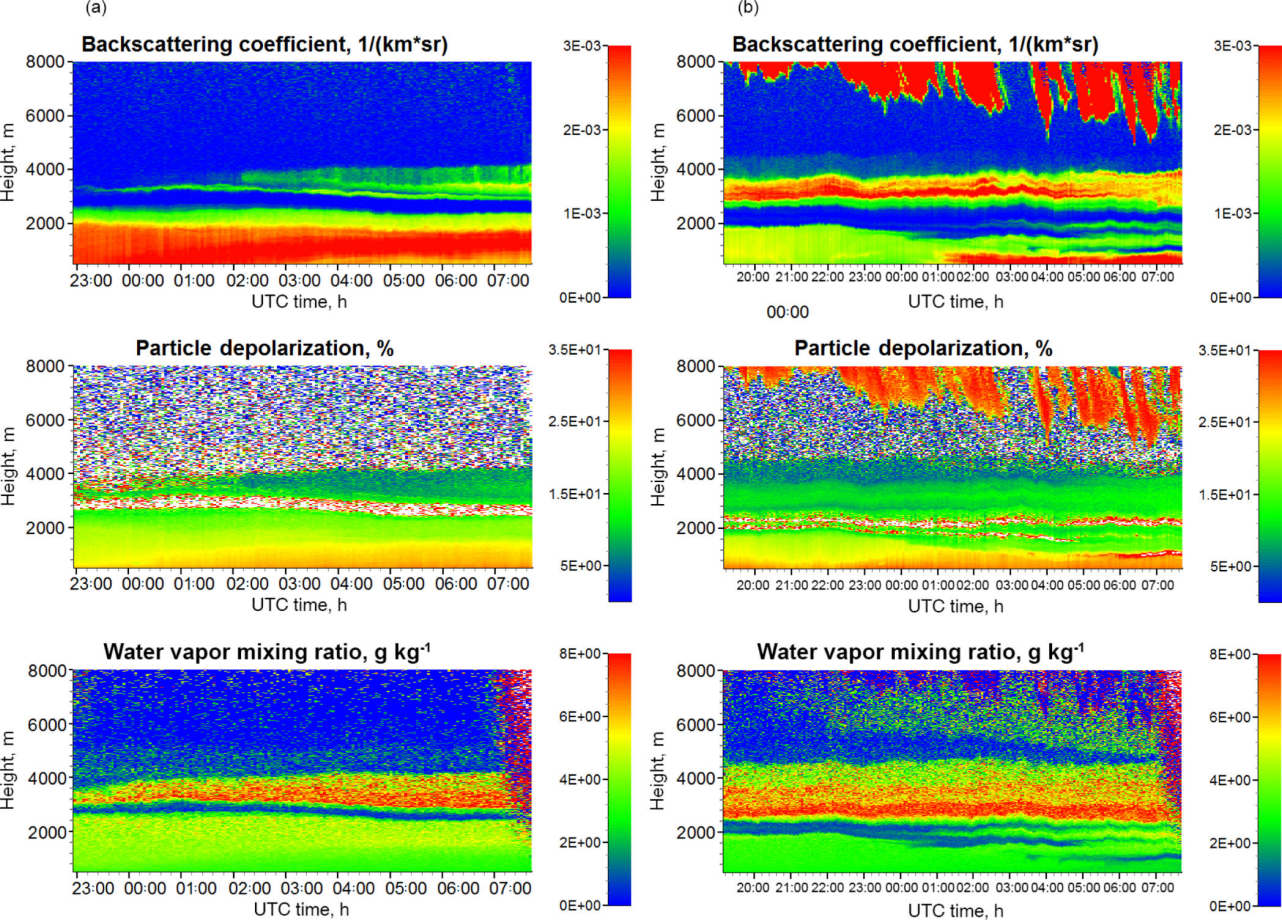
Height–temporal distributions of the backscattering coefficient and particle depolarization ratio at 532 nm together with the water vapor mixing ratio derived from the Raman lidar measurements on the nights of 23–24 (**a**) and 24–25 December 2015 (**b**).

Multiwavelength Raman lidar observations are available for the 23–25 December period only. The height–temporal evolution of the particle backscattering coefficient *β*_532_, depolarization ratio *δ*_532_, and water vapor mixing ratio *w* measured by Raman lidar on the nights 23–24 and 24–25 December 2015 are shown in Fig. 3. Due to the geometrical overlap factor the extinction data can be processed starting from approximately 750 m, and thus plots of all parameters start at this height. The depolarization ratios of pure dust observed during SHADOW are in the 30–35 % range (Veselovskii et al., 2016), while the depolarization ratio of smoke at 532 nm normally is below 10 % (e.g., Tesche et al., 2011; Burton et al., 2015). Hence depolarization measurements provide a convenient way to separate the aerosols into dust and smoke components.

On the night of 23–24 December the dust layer extends up to 2500 m, but with a high depolarization ratio (> 30 %), which is usually associated with pure dust, is observed only below 1000 m, meaning that in 1000–2500 m range the dust is probably mixed with smoke. The optical depth of the elevated smoke layer is rather small on 23–24 December (0.1 at 05:00UTC), but on 24–25 December it increases up to 0.25, making possible the calculation of extinction coefficients from the Raman lidar signals. For analyzing the vertical distribution of smoke and dust particle parameters, we focus on the nighttime measurements of 24–25 December 2015.

**Figure 4.**
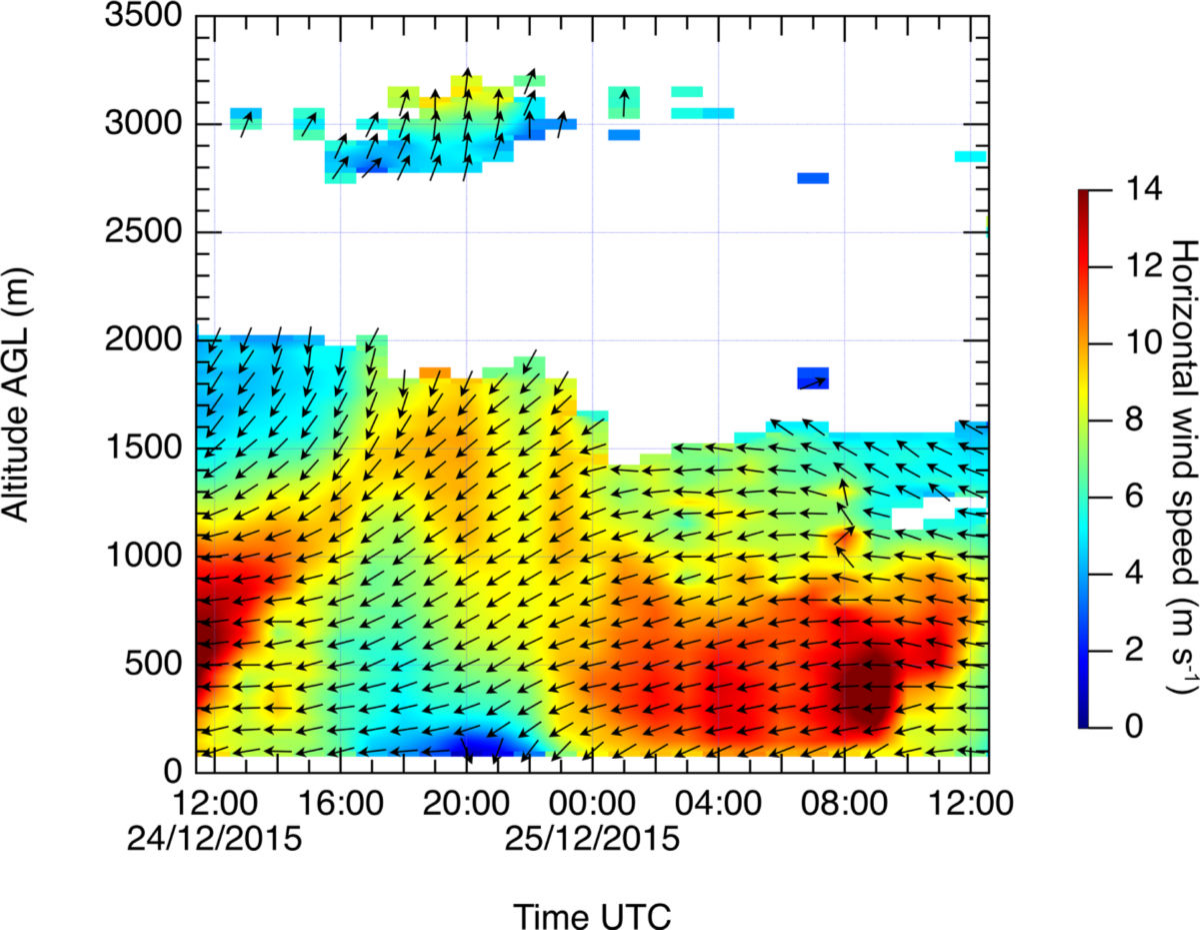
Time–height section of horizontal wind direction (arrows) and wind speed (color map) deduced from Doppler lidar during 24–25 December 2015. Leftward and downward arrows represent, respectively, easterly wind and northerly wind.

**Figure 5.**
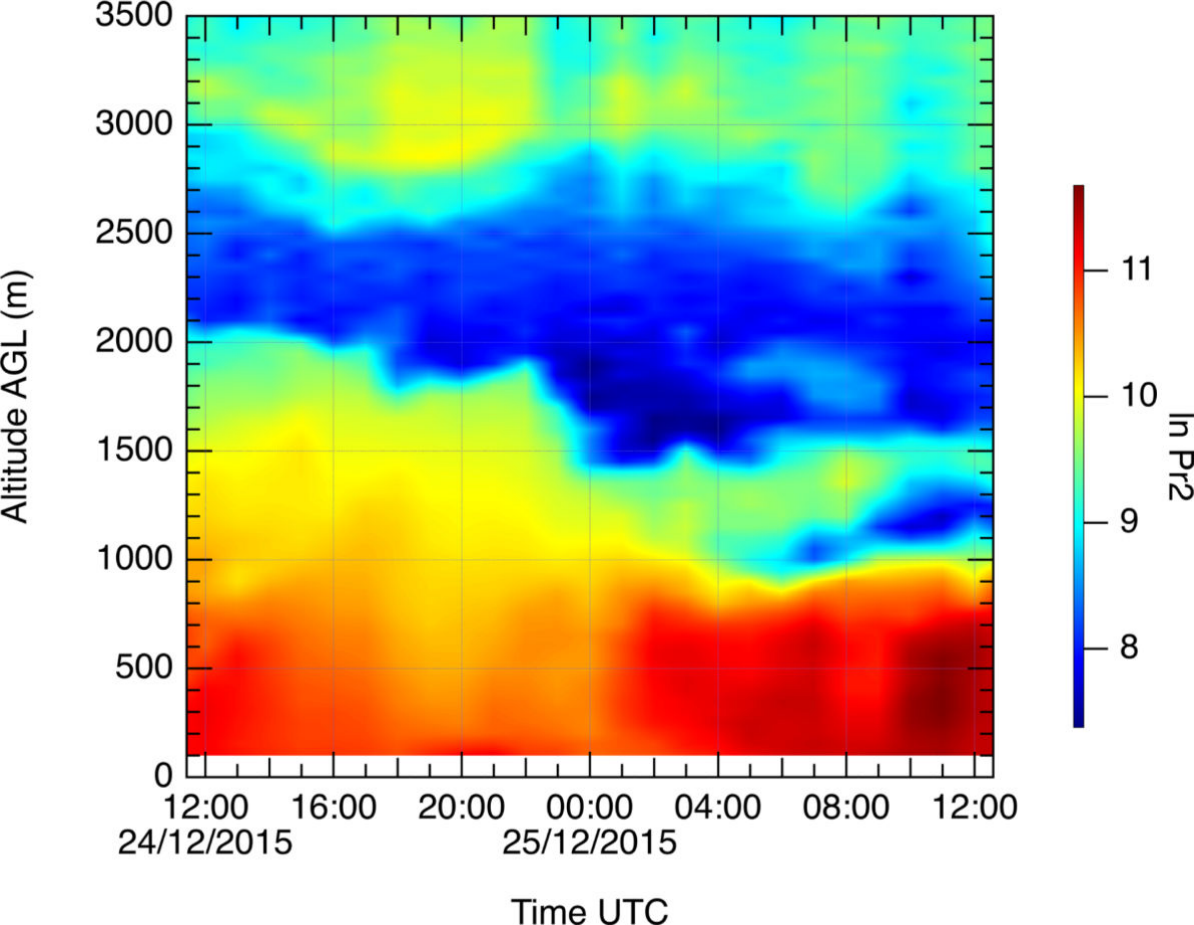
Time–height section of the logarithmic range-corrected lidar signal (in arbitrary units) deduced from the Doppler lidar measurements during the night of 24–25 December 2015.

Figure 4 shows the horizontal wind direction and speed measured by the wind lidar on 24–25 December. The range-corrected signal of the wind lidar can be evaluated starting from 100 m height, and the corresponding height–temporal image is shown in Fig. 5. The wind speed was measured in the dust layer (< 1500 m) for the whole period, but inside the smoke layer the backscatter signal is lower, so the measurements were possible only in the period of 16:00–22:00UTC on 24 December. During 24–25 December 2015, the wind in the low troposphere (< 1500 m) is mainly dominated by the easterly Harmattan continental trades. Deceleration and acceleration of the lower part of the Harmattan (< 1000 m) are observed, respectively, in the beginning of the afternoon and during the night. The vertical profile of the wind speed demonstrates the presence of a low-level jet (LLJ), where the maximum wind speed (jet speed) is located at a height of 350 m (LLJ height) at 01:00 UTC. LLJs are known to contribute to regional horizontal aerosol transport and to increase vertical mixing. Indeed, the LLJ occurrence at 01:00 UTC increases the aerosol loading by transporting desert dust. The corresponding increase of backscattering due to the LLJ at 01:00 UTC on 25 December can also be seen in Fig. 3.

**Figure 6.**
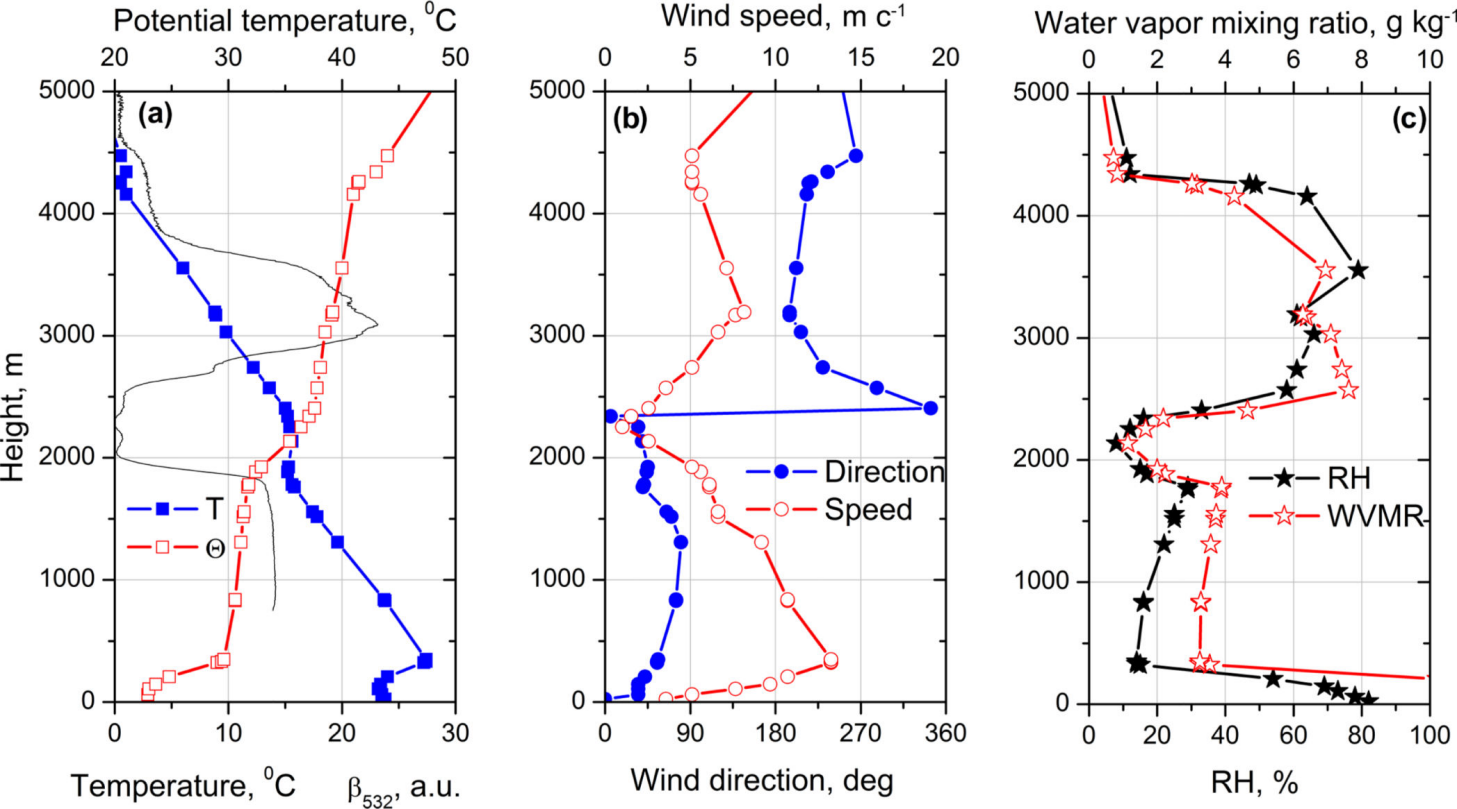
Vertical profiles of (**a**) temperature *T*, potential temperature Θ, (**b**) wind direction and speed, and (**c**) relative humidity (RH) and water vapor mixing ratio (WVMR) measured by the radiosonde in Dakar at 00:00 UTC on 25 December 2015. Solid line in plot (**a**) shows the aerosol backscattering coefficient at 532 nm in arbitrary units measured by the Raman lidar at 21:00 UTC on 24 December.

The vertical profiles of temperature *T*, potential temperature Θ, wind direction and speed, together with relative humidity (RH) and WVMR from radiosonde launched from Dakar at 00:00 UTC on 25 December 2015, are shown in Fig. 6. The profile of wind speed and wind direction obtained from the sonde confirms that the LLJ observed with lidar at Mbour is not a local phenomenon, because it is also observed at Dakar. The vertical profile of the potential temperature suggests that the nocturnal boundary layer top corresponds to the LLJ height. Above 3000 m, the lidar and sonde depict southerly winds which transport the smoke plume. The water vapor mixing ratio increases above 2500 m; as a result the RH in the smoke layer reaches 75 % while in the dust layer RH is below 30 %.

**Figure 7.**
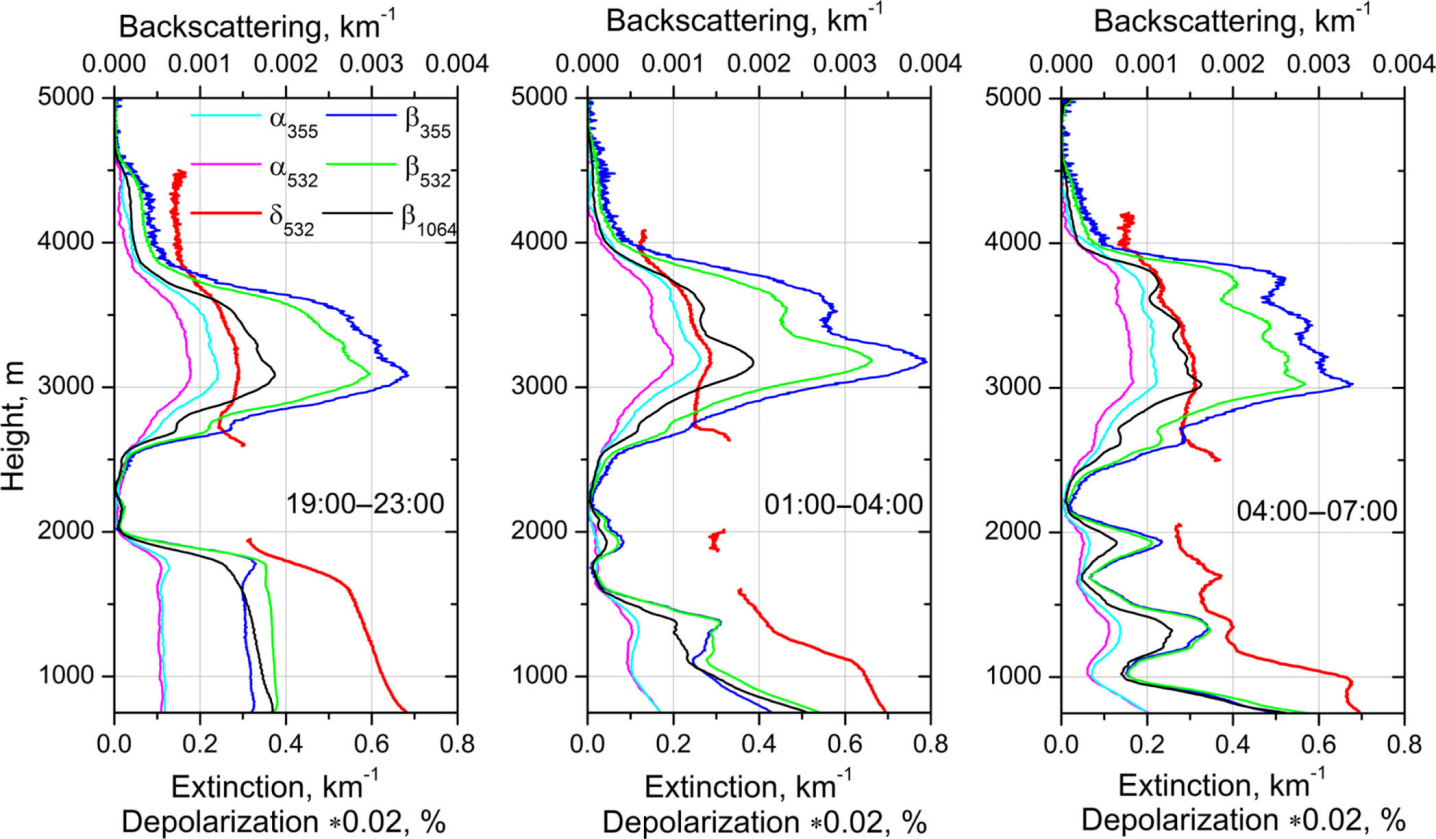
Vertical profiles of the aerosol backscattering (*β*_355_, *β*_532_, *β*_1064_) and extinction (*α*_355_, *α*_532_) coefficients together with the particle depolarization ratio (*δ*_532_) for three temporal intervals: 19:00–23:00, 01:00–04:00, and 04:00–07:00UTC on 24–25 December 2015. The values of *δ*_532_ are multiplied by factor 0.02.

**Figure 8.**
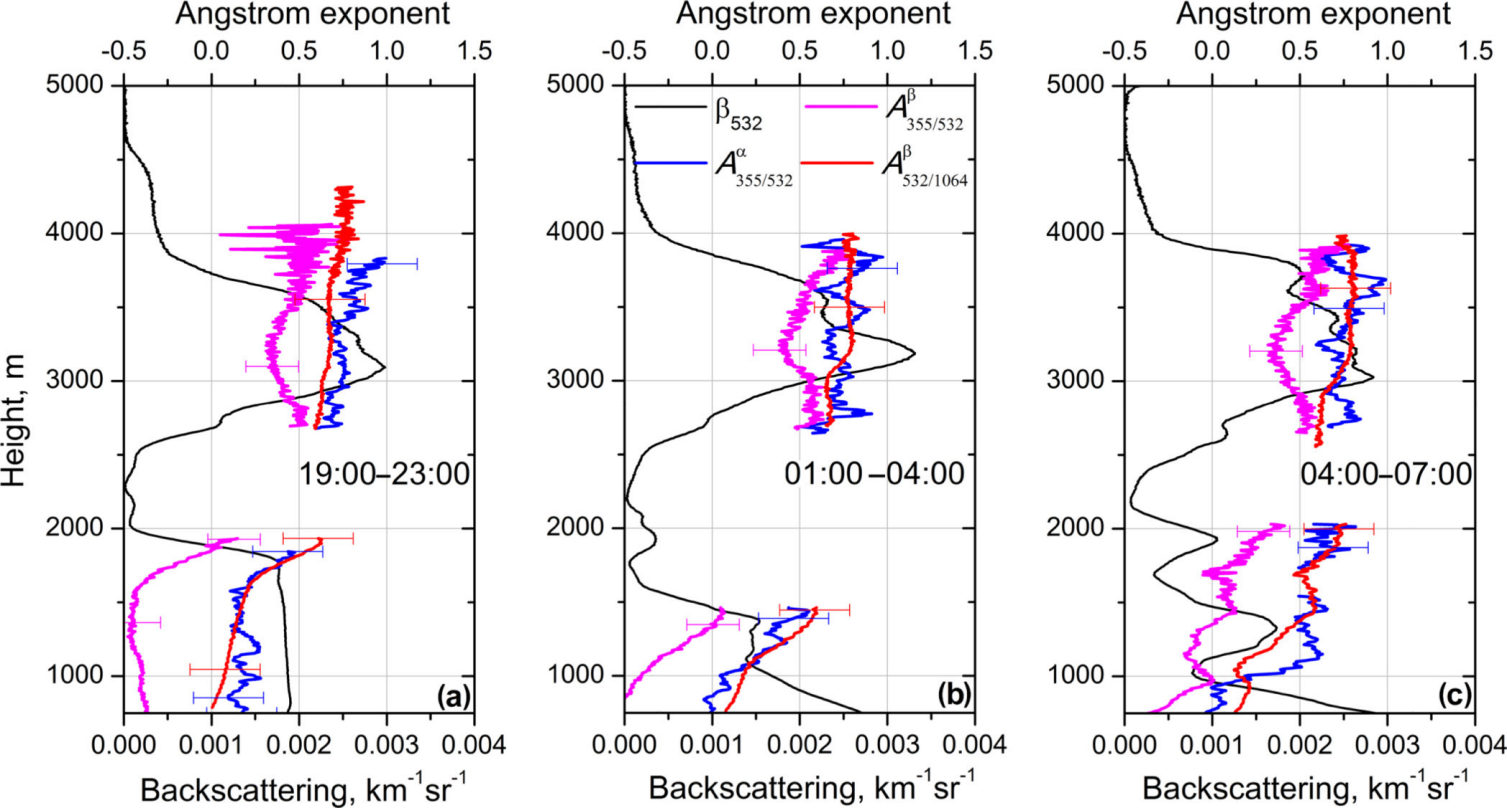
Extinction ($A_{355/532}^{\alpha }$) and backscattering ($A_{355/532}^{\beta },A_{532/1064}^{\beta }$) Ångström exponents together with backscattering coefficient *β*_532_ for the same three temporal intervals as in Fig. 7.

To quantify the vertical distribution of particle parameters, Fig. 7 shows the profiles of backscattering (*β*_355_, *β*_532_, *β*_1064_), extinction (*α*_355_, *α*_532_) coefficients and the particle depolarization ratio (*δ*_532_) derived from Raman lidar measurements for three temporal intervals on the night of 24–25 December: 19:00–23:00, 01:00–04:00, and 04:00–07:00UTC. For the profiles presented, the uncertainty of both *β* and *α* computations is estimated to be below 10 % for the Raman technique and to be below 20% for *β*_1064_ computation by the Klett method. The relative uncertainty of depolarization measurements is below 15 %. The extinction and backscattering Ångström exponents $A_{355/532}^{\alpha },A_{355/532}^{\beta }$, and $A_{532/1064}^{\beta }$ are given by Fig. 8.

For the first temporal interval (Fig. 7a) dust and smoke layers are well separated. Extinction coefficients *α*_355_ and *α*_532_ differ in the smoke layer (*α*_355_ > *α*_532_), but inside the near-surface dust layer (below 1750 m) the extinction values are nearly the same. The depolarization ratio is *δ*_532_ = 35 ± 5 % at 750 m and it gradually decreases with height to 27 ± 4 % at 1750 m. Above that height *δ*_532_ decreases quickly, indicating an increase in the contribution of smoke particles. For the second and third temporal intervals the dust and smoke layers appear to mix, leading to layering in the backscattering coefficient in the 1000–2000 m range. The EAE in this range is increased up to 0.5 (Fig. 8c), indicating that these layers may contain significant amounts of smoke.

The EAE of pure dust observed during SHADOW is slightly negative $A_{355/532}^{\alpha }\approx -0.1$ (Veselovskii et al., 2016). In Fig. 8a the EAE below 1500 m is about 0.2 *±* 0.2, so the dust likely contains some amount of smoke. Values of EAE close to zero are observed in Fig. 8b and c below 1000, where the depolarization ratio increases up to 35 ±5%. Inside the dust layer *β*_355_ < *β*_532_, so the corresponding backscattering Ångström exponent is negative. The negative values of $A_{355/532}^{\beta }$ have been already reported by Veselovskii et al. (2016), where negative BAE was attributed to an increase of the imaginary part of the complex RI at 355 nm compared to 532 nm. In the center of the elevated layer at 3100 m *δ*_532_ = 14 ± 3 %, while at the top of this layer *δ*_532_ decreases to 6 ± 1.5 % (Fig. 8a), indicating a possible presence of dust particles in the center of the elevated layer. The loading of elevated layer with dust particles is supported also by the profiles of $A_{355/532}^{\beta }$: for all three temporal intervals $A_{355/532}^{\beta }$ demonstrates the dip in the center of the elevated layer, while $A_{355/532}^{\alpha }$ and $A_{532/1064}^{\beta }$ do not decrease in the 2500–4000 m range. As mentioned, for pure dust $A_{355/532}^{\beta }$ is negative, so presence of dust in the center of smoke layer should decrease the backscattering Ångström exponent. The presence of dust in the smoke layer is not surprising, because upwelling airflows in forest fires region can lift a significant amount of dust together with biomass burning products (Nisantzi et al., 2014). We should also mention that the spectral dependence of the imaginary part (and thus $A_{355/532}^{\beta }$) depends on the dust origin. In particular, no negative values of $A_{355/532}^{\beta }$ of dust were reported during the SAMUM campaign, so the lidar ratios at 532 nm and 355 nm were close (Tesche et al., 2011).

**Figure 9.**
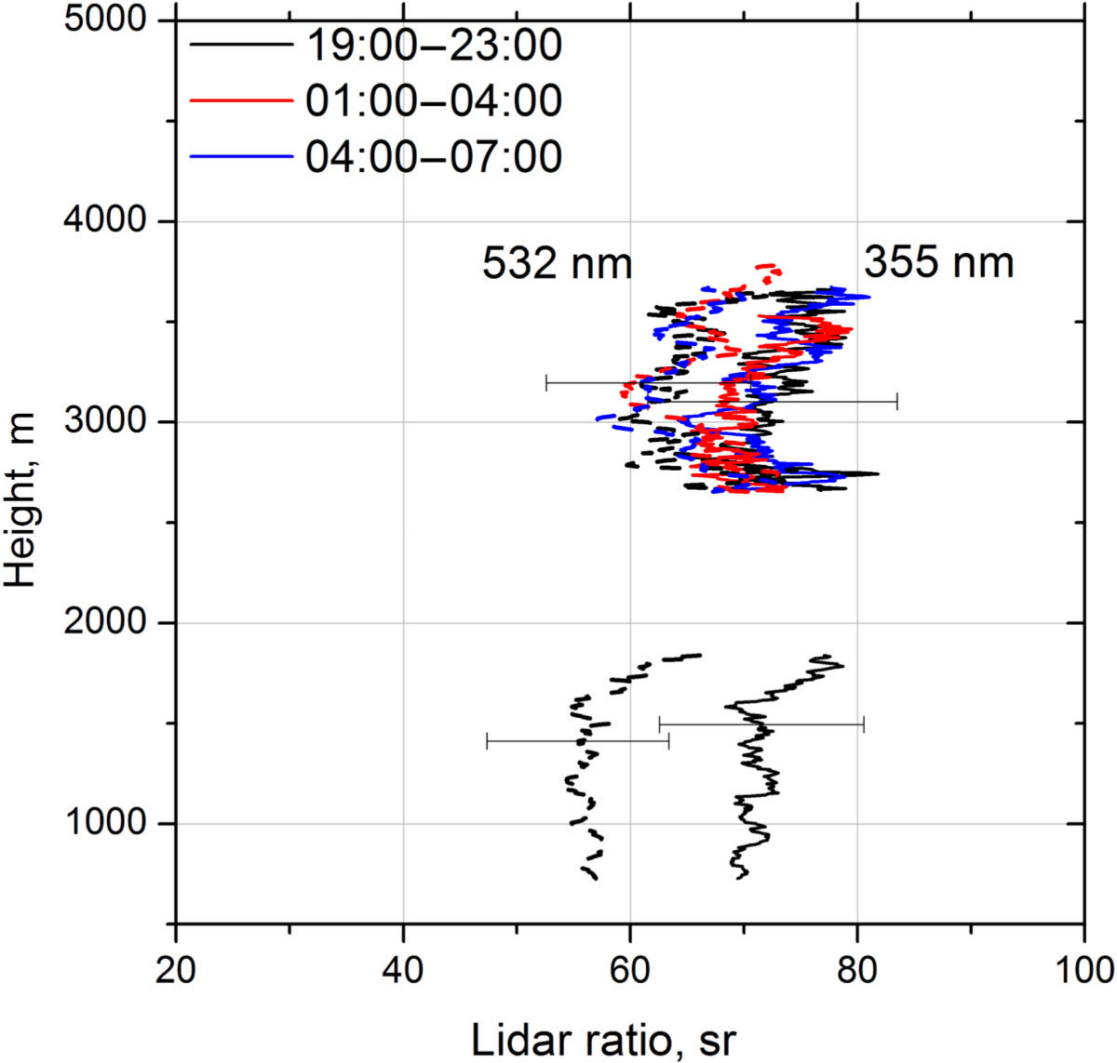
Lidar ratios at 355 nm (solid lines) and 532 nm (dash lines) for three temporal intervals from Fig. 7.

Lidar ratio profiles at 355 and 532 nm, for the same temporal intervals as in Fig. 7, are shown in Fig. 9. The lidar ratios in the dust layer at 532 and 355 nm for 19:00–23:00 UTC period are LR_532_ = 55 ± 8 sr and LR_355_ = 70 ± 10 sr, respectively. At the top of the elevated layer, where the smoke particles are predominant, the lidar ratios for the same period are higher: LR_532_ = 65 ± 10 sr and LR_355_ = 75 ± 11 sr. Due to the presence of dust in the center of the elevated layer, the height dependence of lidar ratios shows a decrease, with a minimum at approximately 3000 m for all three temporal intervals. The decrease is more pronounced at 532 nm because the difference between smoke and dust lidar ratios is larger at this wavelength. The lidar ratios below 2000 m at 01:00–04:00 and 04:00–07:00 UTC become strongly oscillating because of high gradients of backscattering and extinction coefficients at low altitudes and are not shown due to high uncertainties.

**Figure 10.**
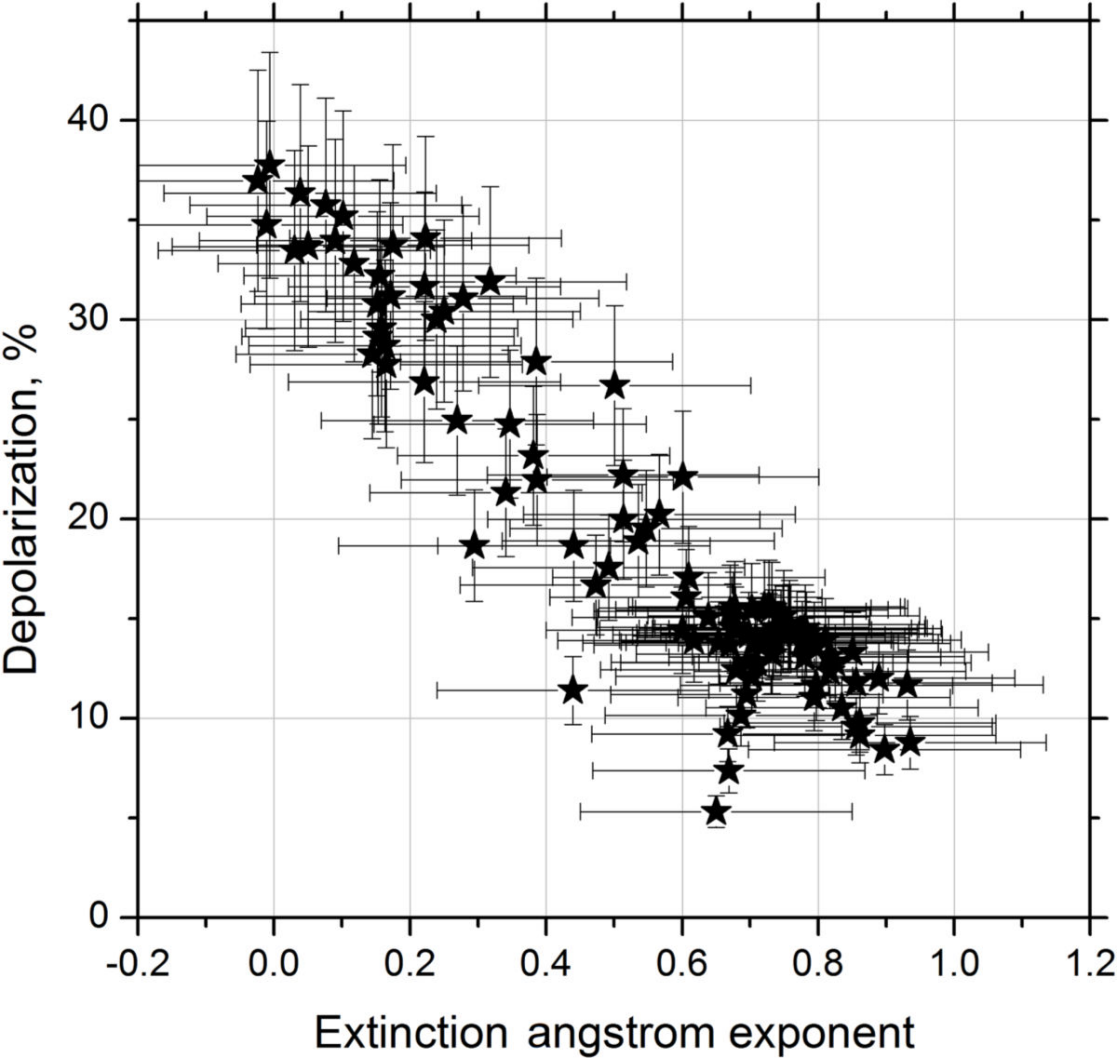
Particle depolarization ratio as a function of the extinction Ångström exponent derived from data shown in Figs. 7 and 8.

Figure 10 shows the dependence of the particle depolarization ratio *δ*_532_ on the extinction Ångström exponent derived from data in Figs. 7 and 8. The depolarization ratio monotonically decreases while EAE rises from 0 to 0.9. Thus observed high values of the depolarization ratio are attributed to big dust particles with EAE close to zero, while small smoke particles are characterized by low depolarization (below 10 %). If depolarization ratios of smoke *δ*^s^ and dust *δ*^d^ are known, the contributions of smoke and dust particles to the total backscattering can be separated *β* = *β*^s^ + *β*^d^ (Sugimoto and Lee, 2006; Tesche et al., 2009; Miffre et al., 2012; David et al., 2013; Burton et al., 2014). Assuming that the depolarization ratios of dust and smoke particles do not change with height the contributions *β*^d^ and *β*^s^ can be calculated as suggested by Tesche et al. (2009):

(6) $$\beta^{\text{d}}=\beta \frac{\left(\delta -\delta^{\text{s}}\right)}{\left(\delta^{\text{d}}-\delta^{\text{s}}\right)}\frac{\left(1+\delta^{\text{d}}\right)}{\left(1+\delta \right)}\;\text{and}\;\beta^{\text{s}}=\beta -\beta^{\text{d}}.$$

In our computations we used values *δ*^d^ = 35 % and *δ*^s^ = 7 %.

**Figure 11.**
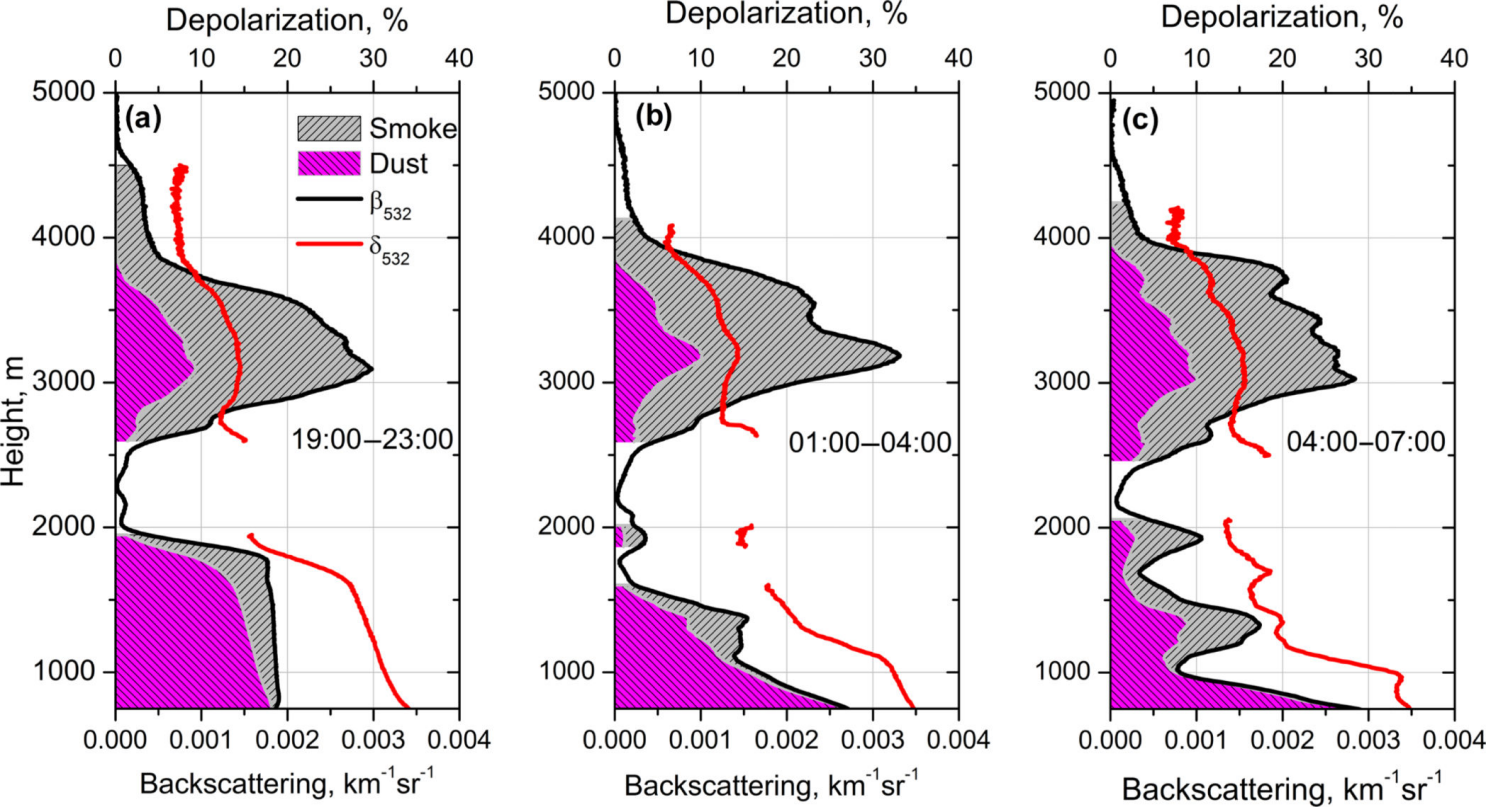
Contributions of dust and smoke to the total backscattering coefficient *β*_532_ together with particle depolarization ratio *δ*_532_ for three temporal intervals on 24–25 December 2015. Magenta and grey regions correspond to dust and smoke contribution to total scattering $\beta_{532}=\beta_{532}^{\text{d}}+\beta_{532}^{\text{s}}$.

The results of the decomposition of *β*_532_ for $\beta_{532}^{\text{d}}$ and $\beta_{532}^{\text{s}}$ components for the same three temporal intervals as in Fig. 7 are shown in Fig. 11. This figure presents the total backscattering coefficient *β*_532_ together with the particle depolarization ratio *δ*_532_. The dust contribution to backscattering is marked with magenta, while the residual backscattering $\beta_{532}-\beta_{532}^{\text{d}}$ is attributed to the smoke and is marked with grey. For the height regions with low backscattering the uncertainty of *β*_532_ is high, so the decomposition for these regions is not shown. The dust is predominant below 1700 m for 19:00–23:00 UTC period, but even the elevated layer contains a significant amount of dust: at 3100 m $\beta_{532}^{\text{d}}\approx 0.3\beta_{532}$. After 01:00 UTC the smoke layers descend (Fig. 3) and their contribution to backscattering becomes significant down to 1000 m height.

## 4 Comparison of lidar measurements with MERRA-2

**Figure 12.**
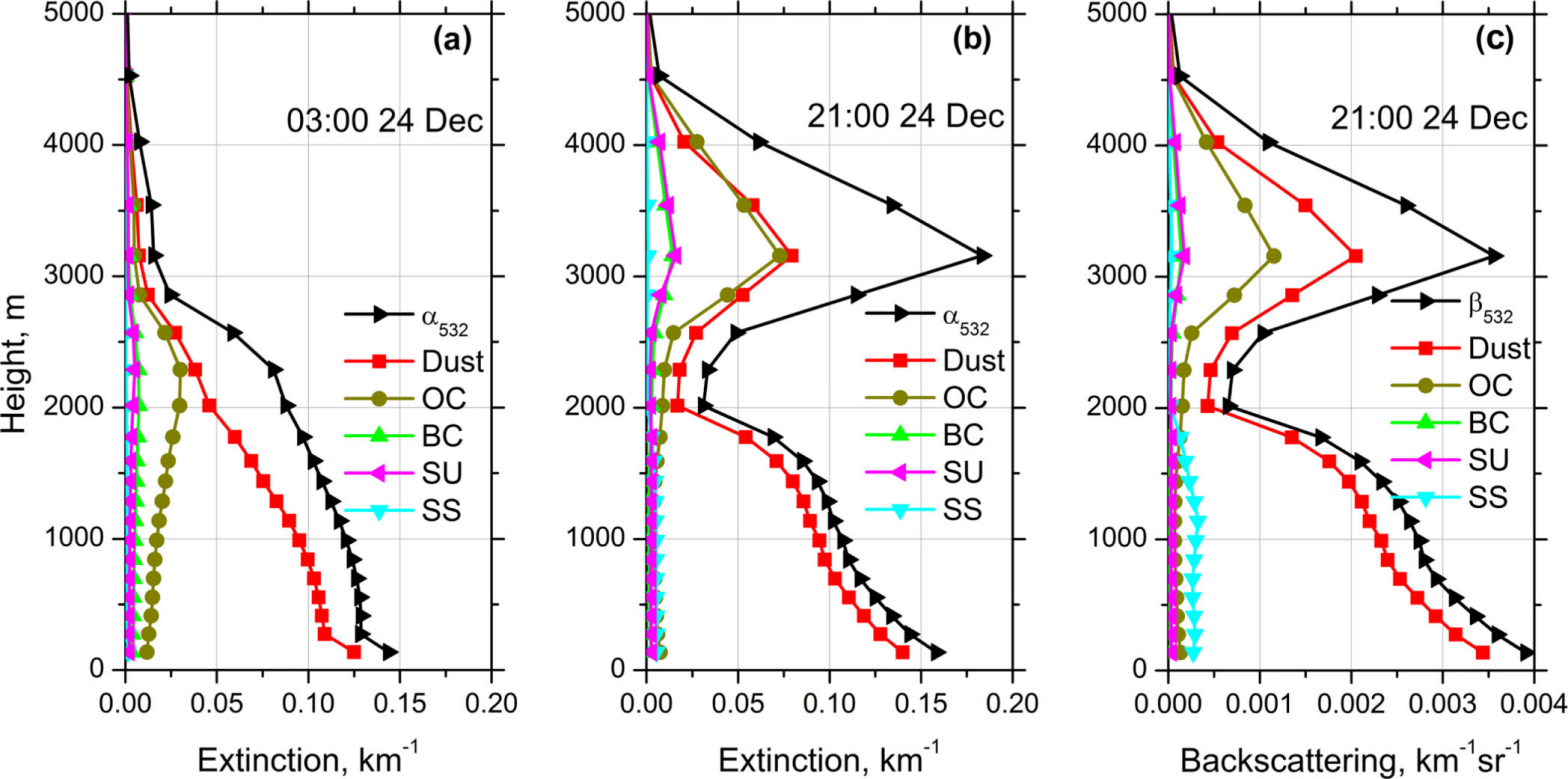
Vertical profiles of extinction coefficients at (**a**) 03:00 UTC, (**b**) 21:00 UTC and (**c**) backscattering coefficients at 21:00 UTC on 24 December 2015 from MERRA-2 model at 532 nm. Profiles are given for five aerosol components: dust, black carbon (BC), organic carbon (OC), sea salt (SS), and sulfates (SU), together with total extinction *α*_532_ and backscattering *β*_532_.

MERRA-2 provides the vertical distribution of mass mixing ratios of five aerosol components, so for each of these components the extinction, backscattering coefficients, and depolarization ratios can be calculated. The vertical profiles of extinction coefficient of dust, BC, OC, SS, and SU, together with total extinction *α*_532_, are shown in Fig. 12 for 03:00UTC and 21:00UTC on 24 December 2015. At 03:00 UTC the aerosol is localized below 3000 m. Dust extinction is predominant, but contribution of OC to the total extinction coefficient rises with height,, reaching maxima at 2250 m. The presence of a significant amount of OC agrees with the low values of depolarization ratio above 1500 m for this temporal interval in Fig. 3.

At 21:00 UTC an elevated layer with a maximum of extinction at 3150 m is observed (Fig. 12b). In this layer OC and dust provide similar contributions to extinction (about 40% at 3150 m height). From the results shown in Fig. 11a we can estimate the contribution of dust to *α*_532_ in the center of the elevated layer as 30 % (by assuming the dust lidar ratio LR_532_ = 55 sr), so the measured and simulated dust contributions are in good agreement. Below 1750 m the dust is the main contributor to the extinction coefficient providing 88% of *α*_532_ at 1000 m (Fig. 12b). The observed dust contribution to *α*_532_ at the same height is about 90 % (Fig. 11a), which again shows good agreement between the model and measurements. Total contribution of BC and SU to extinction is below 20 % in the elevated smoke layer, and in the near-surface dust layer their contribution is negligible. The extinction coefficients can be recalculated to the backscattering using model lidar ratios of the aerosol components. Figure 12c shows the profiles of backscattering coefficients at 532 nm computed for the same temporal interval as in Fig. 12b. The simulation of the backscattering coefficient is more challenging than that of extinction, because backscatter depends more strongly on the particle morphology and RI. A detailed comparison of measured and modeled profiles of backscattering coefficients will be performed later in this section.

**Figure 13.**
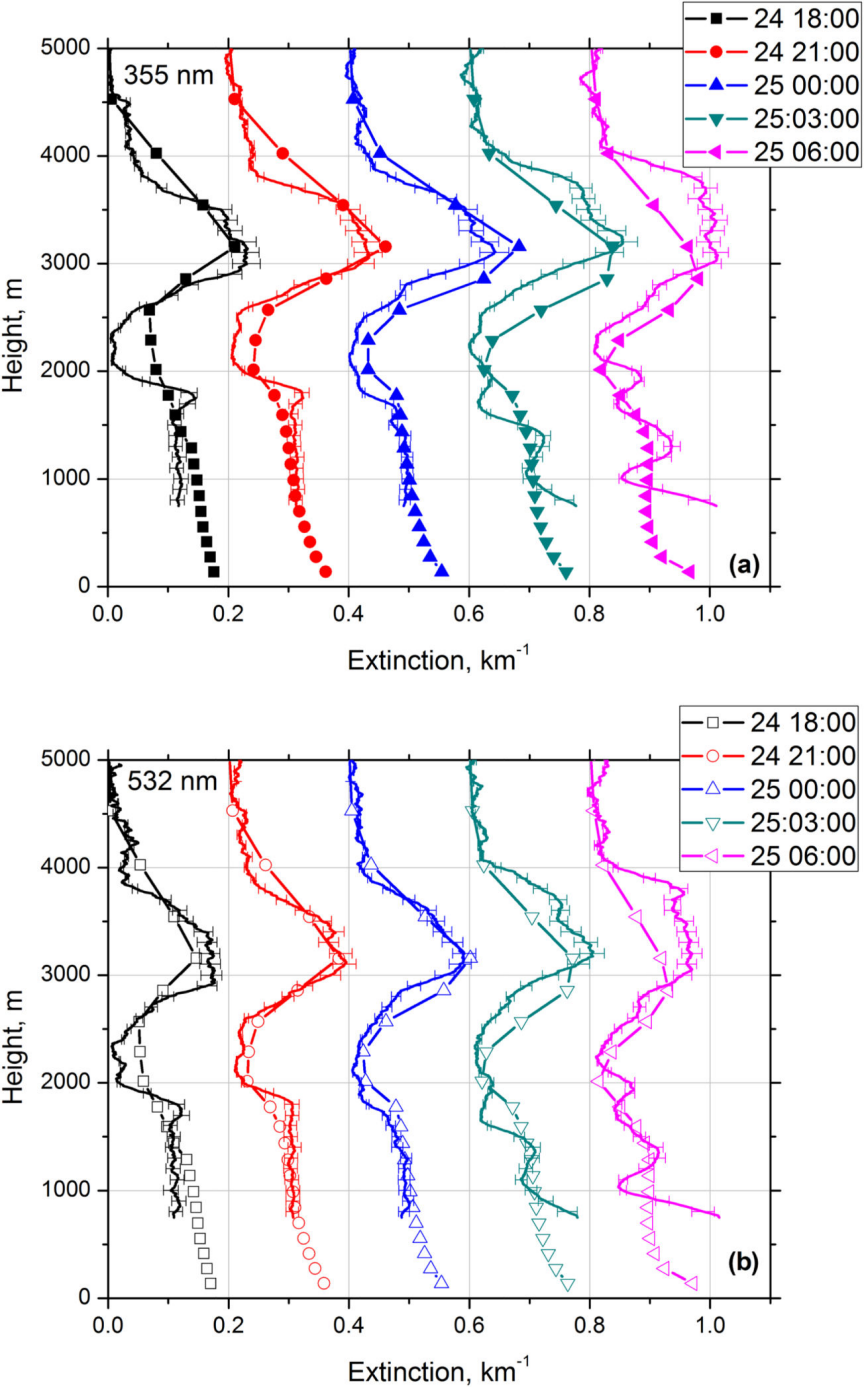
Comparison of extinction profiles at (**a**) 355 nm and (**b**) 532 nm derived from Raman lidar measurements (line) and modeled by MERRA-2 (line + symbols) on the night of 24–25 December 2015. Model profiles are provided at 18:00, 21:00, 00:00, 03:00, and 06:00 UTC. The lidar measurements are given for temporal intervals centered at 19:00, 21:00, 00:00, 03:00, and 06:00 UTC. For each profile, 2 h of measurements are averaged. The profiles are shifted relatively to each other by 0.2 km^−1^.

As mentioned, the comparison of model and observed values is more straightforward for extinction coefficients. Figure 13 shows the time series of extinction profiles at 355 and 532 nm modeled for the night of 24–25 December 2015 at 18:00, 21:00, 00:00, 03:00, and 06:00UTC. The profiles are shifted relative to each other by 0.2 km^−1^. For comparison, the same figure presents the profiles of extinction coefficients derived from Raman lidar measurements. The model reproduces well the location of the elevated smoke layer as well as the top of the near-surface dust layer. However, the model does not resolve the oscillations of extinction profile below 2000 m at 03:00 and 06:00UTC on 25 January.

To quantify the difference between the measured (*α*^meas^) and modeled ($\alpha^{⊥}$) extinction coefficients the difference $\Delta \alpha =\alpha^{\text{meas}}-\alpha^{⊥}$ was calculated. The statistical analysis of the frequency distribution of Δ*α* for all five profiles in Fig. 13 shows that at 355 nm the mean value of Δ*α* is −0.01 km^−1^ and SD of 0.042 km^−1^. With typical values of extinction coefficient in elevated smoke layer and near-surface dust layer being on the order of 0.2 km^−1^, the relative difference of modeled and measured extinction is estimated to be below 25 % for the time period considered. The results of statistical analysis for *α*_532_ nm are similar.

**Figure 14.**
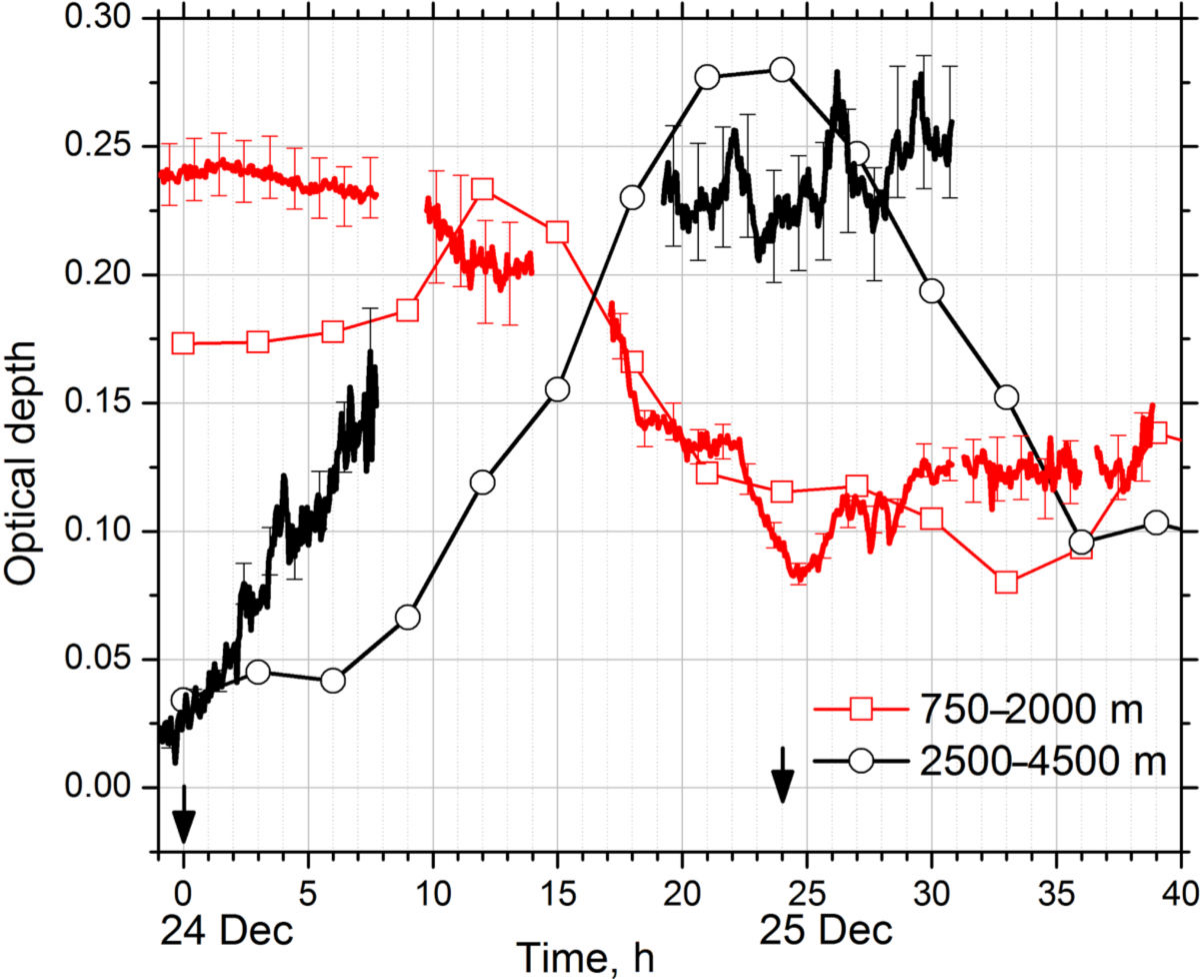
Aerosol optical depth at 355 nm on 23–24 December 2015 obtained from MERRA-2 (line + symbols) and from the Raman lidar measurements (solid lines). The results are given for two height intervals: 750–2000 m (red) and 2500–4500 m (black). Zero of timescale corresponds to 00:00 UTC on 24 December.

To analyze how well the model reproduces the temporal variations of aerosol optical depth, Fig. 14 presents AODs at 355 nm on 23–24 December 2015 for two height intervals: 750–2000 and 2500–4500 m. The first interval corresponds to the near-surface dust layer, while the second interval corresponds to the elevated smoke layer. The AOD is calculated from the Raman backscatter channel, and in the daytime measurements could be processed only in the dust layer due to enhanced background noise. Thus daytime measurements in the elevated smoke layer are not plotted. The time of the appearance of the smoke layer is well represented in the model results (about 00:00 UTC on 24 December), but the lidar-derived AOD of this layer increases rapidly from the first appearance of the layer, while in the model the rapid increase in AOD growth starts approximately 5 h later. The model predicts that the maximum value of AOD in the smoke layer (0.27) is reached at 20:00–24:00 UTC interval, which reasonably agrees with observations: mean value of measured AOD for this interval is 0.23 ± 0.02. After midnight the modeled AOD of the smoke layer decreases quickly, while lidar measured AOD stays about 0.25. The measured AOD of the near-surface layer agrees with the model. The observed AOD exceeds the model values in the beginning (at 00:00 UTC on 24 December measured and modeled AODs are 0.24 and 0.175, respectively), but after 10:00 UTC the values are in better agreement. Thus, we can conclude that the model reproduces the temporal variability of AOD in the dust and smoke layers.

The agreement between modeled and observed extinction profiles provides an opportunity to test how well the backscattering coefficients can be modeled. Simulation of backscattering coefficients is especially challenging for dust for several reasons. First of all, we are not confident in the accuracy of the presumed scattering phase function in the backward direction. Second, the backscattering coefficient strongly depends on the particle RI, in particular on the imaginary part, which may vary over a wide range depending on dust origin. The in situ ground measurements in West Africa, performed during the SAMUM field campaign, demonstrate that the mean value of *m*_I_ for dust episodes is about 0.003 at 532 nm and 0.02 at 355 nm. However, deviation from these mean values for every individual measurement can be significant (Müller et al., 2009; Kandler et al., 2011; Ansmann et al., 2011). The imaginary part of RI of dust in the model is assumed to be 0.007 at 355 nm, following previous OMI data analysis (Torres et al., 2007), and 0.0025 at 532 nm.

**Figure 15.**
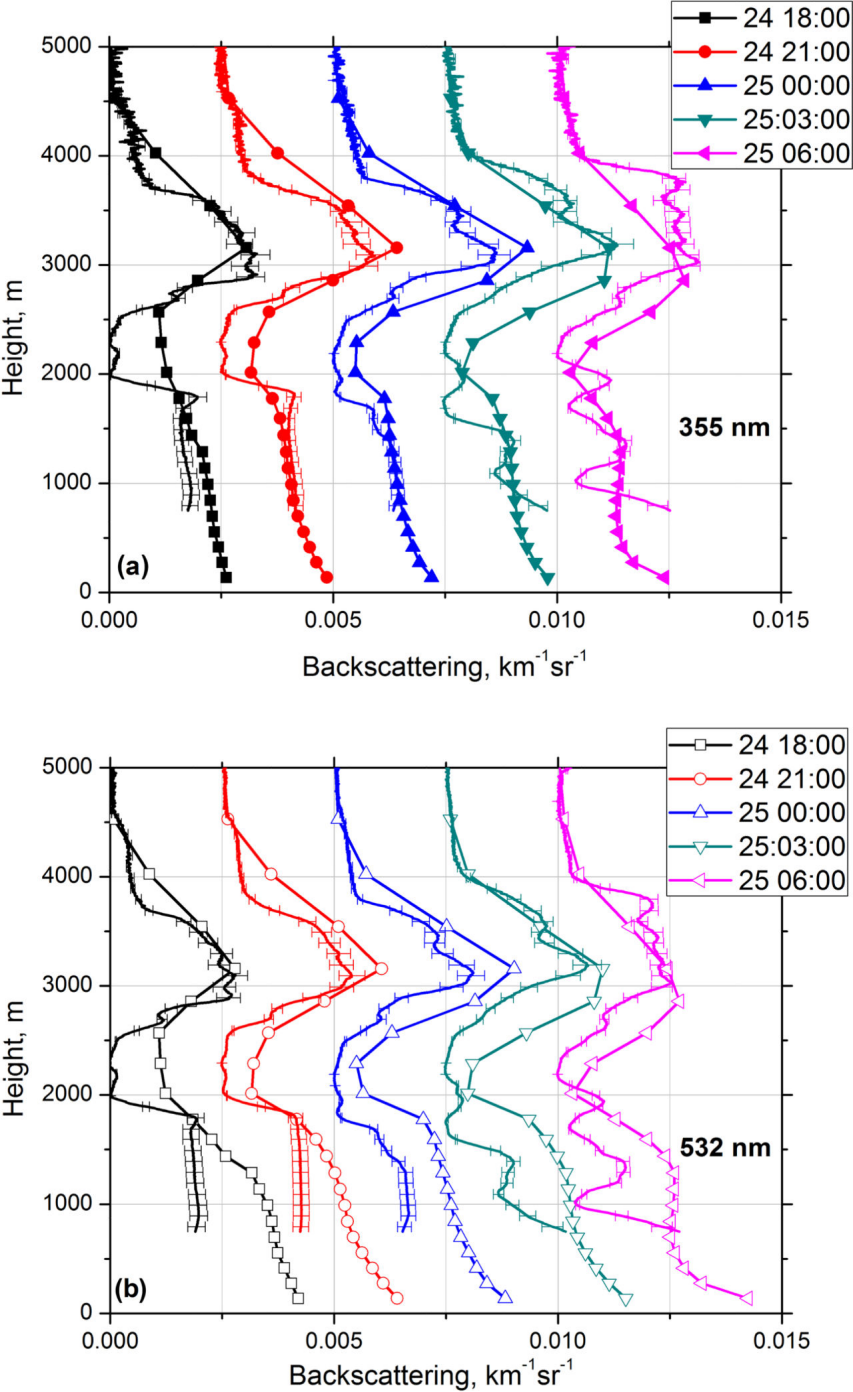
Backscattering coefficients at (**a**) 355 nm and (**b**) 532 nm measured by Raman lidar (solid line) and modeled by MERRA-2 (line + symbols) on the night of 24–24 December 2015. Profiles are shifted relatively to each other by 0.0025 km^−1^ sr^−1^. The temporal intervals are the same as in Fig. 13.

**Figure 16.**
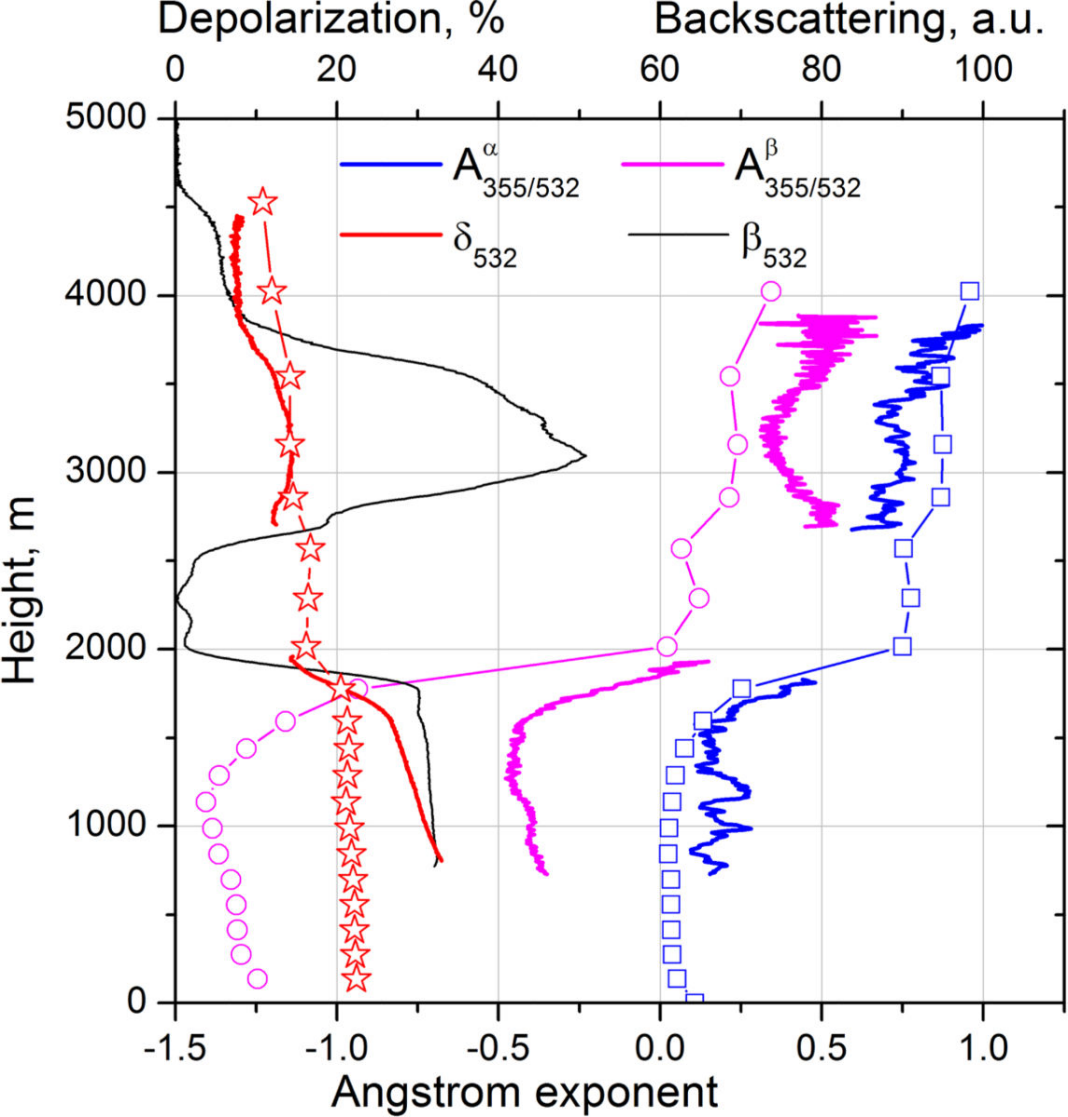
Extinction ($A_{355/532}^{\alpha }$) and backscattering ($A_{355/532}^{\beta }$) Ångström exponents together with the particle depolarization ratio *δ*_532_ obtained from lidar measurements (line) and from MERRA-2 modeling (line + symbols). Lidar data are averaged over the 19:00–23:00 UTC period while model data are given for 21:00 UTC.

Figure 15 shows measured and modeled backscattering coefficients at 355 and 532 nm for the same five temporal intervals as in Fig. 13. At 355 nm the modeled and measured values agree for both the smoke and dust layers. However, at 532 nm the aerosol backscattering coefficients agree only inside elevated layer, while below 1750 m the modeled *β*_532_ significantly exceeds the measured values. As mentioned, the modeled lidar ratio LR_532_ for the mixture is close to 40 sr at 1000 m, while the measured lidar ratio in the near-surface dust layer is 55 ± 8 sr. The reason for this disagreement could be that the assumed imaginary part of the RI for dust (0.0025 at 532 nm) is too low. Recall, however, that we cannot determine the imaginary part of the RI for dust by simply adjusting the modeled lidar ratio to the measured one, because the lidar ratio depends on several factors besides *m*_I_, such as the PSD and the aspect ratio of the ellipsoids used in the model. It is possible that the PSD in the model is weighted too much toward fine-mode dust. The modeled and measured particle intensive parameters, such as extinction $A_{355-532}^{\alpha }$ and backscattering $A_{355-532}^{\beta }$ Ångström exponents and the particle depolarization ratio *δ*_532_, are shown in Fig. 16. The measurements are averaged over 19:00–23:00 UTC interval while modeled values are given for 21:00 UTC. The model reproduces well the observed vertical distribution of $A_{355-532}^{\alpha }$ in both the dust and the elevated layer. As follows from Fig. 7a, inside the dust layer *β*_355_ < *β*_532_, so the corresponding $A_{355-532}^{\beta }$ is negative with a minimum value of about −0.4. The model predicts values of $A_{355-532}^{\beta }$ as low as −1.4. The modeled BAE is sensitive to the choice of the imaginary part of RI at 355 and 532 nm and, as mentioned, the chosen *m*_I_(532) = 0.0025 may be too low for this episode. In the elevated layer the modeled $A_{355-532}^{\beta }$ is close to the observed one. The modeled BAE has no minimum in the center of the elevated layer, because the modeled ratio of dust and OC aerosol concentrations shows only a small variation throughout the elevated layer.

The model reproduces reasonably well the depolarization in the elevated layer, but inside the dust layer the modeled *δ*_532_ is significantly lower than what is observed (22 % compared to 35 %). This problem is well known: the spheroidal model underestimates the depolarization ratio when typical dust PSD and complex RI are used (Veselovskii et al., 2010; Wiegner et al., 2009; Müller et al., 2013; Nowottnick et al., 2015).

**Figure 17.**
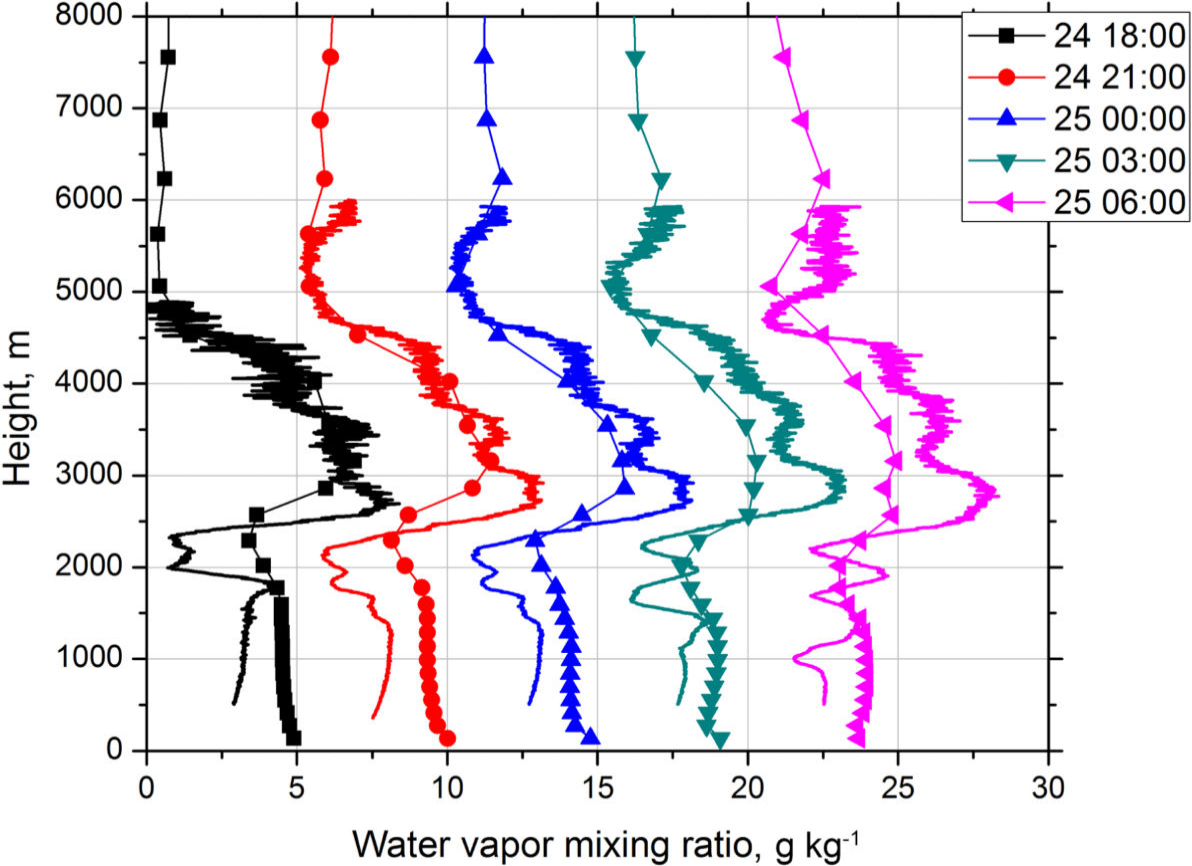
Water vapor mixing ratio derived from Raman lidar measurements (solid line) and obtained from the model (line + symbols) on the night of 24–25 December 2015. Temporal intervals are the same as in Fig. 13. The profiles are shifted relatively each other by 5 g kg^−1^.

One of the MERRA-2 data products is WVMR, which helps to identify atmospheric parcels, is critically important for determining atmospheric stability, and serves as the source of water for aerosol hygroscopic growth. Figure 17 shows five model profiles of WVMR together with the results of Raman lidar measurements for the same temporal intervals as in Fig. 13. The model reproduces rather well the WVMR profile inside the elevated layer (2500–4500 m) on 24 December, though on 25 December the modeled values in this range are lower than the observations. In the near-surface dust layer, the deviation of modeled values from the measurements is larger. Statistical analysis of the deviation of modeled values from lidar measurements for all five profiles shows that mean difference is 0.04 g kg^−1^ with SD of differences of 1.6 g kg^−1^. Thus in the elevated layer, where WVMR is approximately 8 g kg^−1^, the agreement is quite good, but in the dust layer, which is characterized by low water vapor content (below 4 g kg^−1^), the difference may be up to 40 %.

## 5 Inversion of lidar measurements to particle microphysical properties

In the previous section, as validation of the model output we compared the modeled aerosol optical parameters, such as extinction, backscattering coefficients, and depolarization ratio with the values derived from lidar measurements in a straightforward way. The comparison of particle microphysical properties such as volume, effective radius, and complex RI, however, is not straightforward, since it needs inversion of the measurements and requires additional assumptions. In the case of dust particles the inversion becomes especially challenging for the following reasons:

– The size distribution of dust contains a strong coarse mode with particle radii extending up to ~ 15 pm, and the estimation of properties for such big particles is difficult since measurements are only performed in the wavelength range 0.355–1.064 μm.
– The inversions have to consider the RI as spectrally independent. In fact, the imaginary part of the dust RI is spectrally dependent with a strong enhancement at 355 nm compared to 532 nm.
– The dust particles are not spherical and so the application of Mie formulas for the forward modeling results in errors in computing the scattering phase function.

Regarding the shape issue, one of the ways to mimic the scattering properties of dust particles is to use the model of randomly oriented spheroids (Mishchenko et al., 1997; Dubovik et al., 2006). The implementation of this model for inversion of dust lidar measurements is described in Veselovskii et al. (2010, 2016) and Müller et al. (2013). This algorithm was used also for inversion of our 3*β* + 20*α* observations. The range of particle radius in the inversion has been set to a minimum and maximum of 0.075 and 15 μm, respectively. The real part of RI was allowed to vary in the range 1.35–1.65, while the imaginary part varied in the range 0–0.02. The RI was assumed to be spectrally independent. The effects of a possible spectral dependence of the imaginary part of RI were considered in Veselovskii et al. (2016).

**Figure 18.**
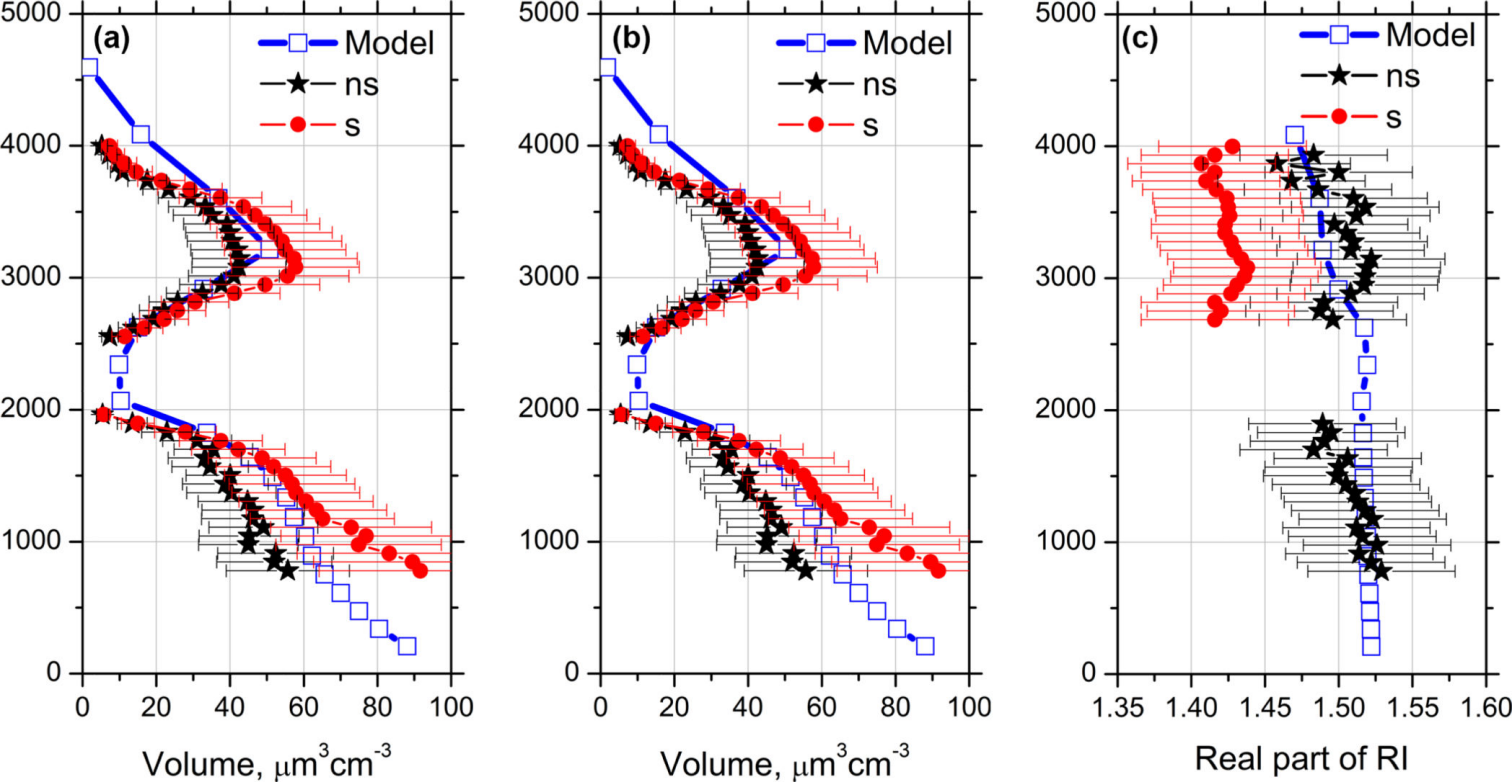
Profiles of (**a**) effective radius, (**b**) particle volume, and (**c**) real part of the refractive index on 24 December 2015 retrieved from 3*β* + 2*α* lidar measurements shown in Fig. 7a (solid symbols) and provided by MERRA-2 for 21:00 UTC (open symbols). Inversion of lidar measurements was performed in assumption of spherical particles (s) and using the model of spheroids (ns).

Profiles of the effective radius, volume density, and real part of the RI retrieved from optical measurements in Fig. 7a are shown in Fig. 18. The inversion was performed for two cases, with the assumption of all spherical particles or all spheroids. A realistic solution (for the mixture of spherical and non-spherical particles) should be closer to spheroids in the dust layer, while in the elevated layer (where depolarization ratio is below 15 %) it should be closer to the results obtained with spheres. The model results provided by MERRA-2 are shown on the same plot. The effective radius and volume density obtained in assumption of spherical particles are always higher than the values obtained with spheroids. The modeled effective radius at 1100 m height is 1.1 μm, which is close to *r*_eff_ = 0.95 ± 0.3 μm obtained from lidar measurements using the spheroids model. Comparing the lidar retrievals with model in the dust layer, we should keep in mind that inside 1500–2000 m height range the dust particles are mixed with biomass burning products; thus the use of only spheroids in retrieval underestimates the effective radius and volume. Moreover, accounting for the spectral dependence of the imaginary part of the dust may additionally increase the retrieved values of *V* and *r*_eff_ by factor 1.2–1.3 (Veselovskii et al., 2016).

Lidar-derived effective radius in the elevated layer at 3000 m is approximately 0.4 and 0.5 μm when spheroids and spheres are used, respectively, while the modeled value is 0.3 μm. The reason for the lower value of modeled effective radius is the contribution of black carbon, which is characterized by small size and relatively low hygroscopic growth. Recall that in the inversion of lidar measurements, the smallest radius considered is 0.075 μm. Modeled values of the volume density agree well with lidar retrievals in both dust and elevated layers.

The estimation of the real part of RI from lidar measurements is sensitive to the type of kernel functions chosen for retrieval. In the regularization algorithm the treatment of dust particles as spheres strongly underestimates *m*_R_ (Veselovskii et al., 2010), so results obtained with spheres in the dust layer are not shown in Fig. 18c. At 1000 m the *m*_R_ retrieved with spheroids is 1.52 ± 0.05, which agrees well with the modeled value. Inside the elevated smoke layer, where fine-mode particles predominate, the application of spheroids overestimates *m*_R_. The lidar-derived real part of RI at 3000 m is 1.43 ± 0.05 for spheres and 1.51 ± 0.05 for spheroids, so we expect that the true value would lie within this. The simulated value of *m*_R_ = 1.50 in the elevated layer is quite high, which is again the result of BC contribution.

**Figure 19.**
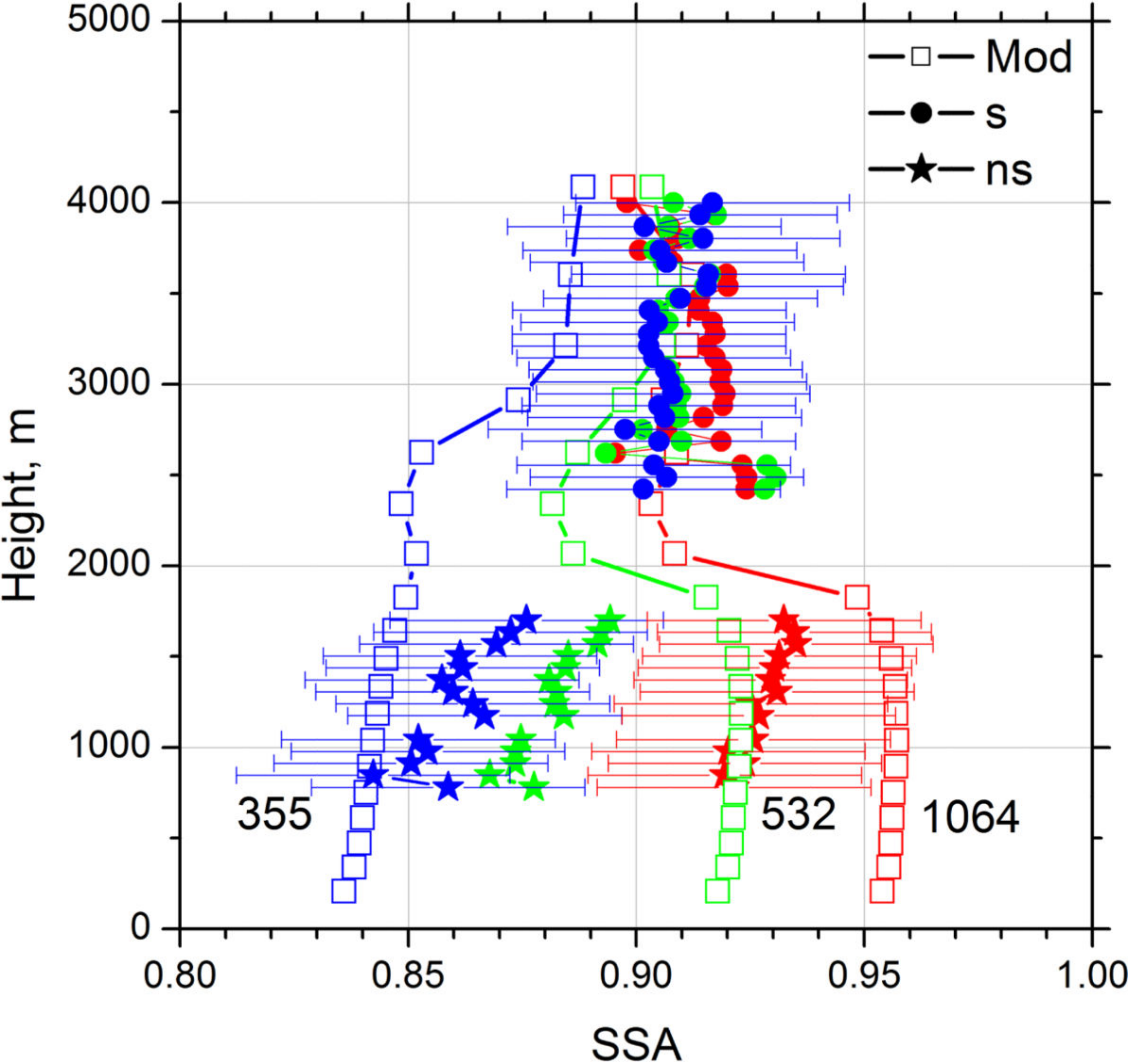
The single scattering albedo at 355 nm (blue), 532 nm (green), and 1064 nm (red) on 24 December 2015 retrieved from 3*β* + 2*α* lidar measurements shown in Fig. 7a (solid symbols) and provided by the MERRA-2 model for 21:00 UTC (line + open symbols). For inversion of lidar data the spheroids (ns) were used below 2000 m and spheres (s) above 2000 m.

The single scattering albedo (SSA) is one of the key parameters to be retrieved and conclusions about the potential of the multiwavelength lidar method strongly rely on its ability to profile SSA. Figure 19 shows SSA at 355, 532, and 1064 nm. As mentioned, the spectral dependence of *m*_I_ was not accounted for and the algorithm retrieves an average value of the imaginary part over the interval of 355–1064 nm. In particular, for dust and OC the imaginary part is underestimated at 355 nm and overestimated at 532 and 1064 nm. As a result, in the dust layer the retrieved SSA exceeds the model values at 355 nm, while at 532 nm and 1064 the situation is opposite. Still at a height of 1000 m, the difference between modeled and lidar-derived SSAs is below 0.04 for all wavelengths. In the elevated layer, where the spectral dependence of *m*_I_ is less pronounced, the simulated and retrieved SSAs agree well with a corresponding difference of less than 0.02.

## 6 Summary and conclusion

The synergy of lidar observations with the aerosol transport model has a great potential to improve the characterization of aerosols properties, and as a first step in such synergy one has to demonstrate how well observations and models agree and describe the same aerosol scenario. For that we have considered a smoke–dust episode over West Africa to compare the vertical profiles of particle parameters modeled by MERRA-2 and retrieved from Raman lidar measurements. In the case selected, the simultaneous presence of the dust and smoke layers resulted in significant height variation of particle parameters, providing a good opportunity to test the models’ capability to reproduce complicated vertical structure. Modeled and observed vertical profiles of *α*_355_ and *α*_532_ show good similarity: MERRA-2 provides the correct location of both the near-surface and elevated layers.

The modeling of the dust lidar ratio is challenging due to irregularity of the particles shape and due to the spectral dependence of the imaginary part of the RI. The *m*_I_ can change significantly for dust of different origin and this variability may be accounted for in future model developments. The modeled at 355 nm the lidar ratio of 65 sr in the near-surface dust layer is close to the observed value (70 ± 10 sr). At 532 nm, however, the simulated dust lidar ratio (about 40 sr) is lower than measurements (55 ± 7 sr). This discrepancy may be an indication that *m*_I_ of dust during the episode considered is higher than the value assumed in the model. Another possible explanation is that the model PSD is too much weighted toward fine-mode dust. The measured lidar ratios at the top of the elevated layer, where smoke particles are predominant, are LR_355_ = 75 ± 11 sr and LR_532_ = 70 ± 10 sr, which is close to the corresponding model values for organic carbon of 71 and 66 sr, respectively.

MERRA-2 predicts the existence of a significant amount of dust in the elevated smoke layer, and the high values of observed depolarization ratio agree with this prediction. The existence of minima of $A_{355/532}^{\beta }$ in the center of the elevated layer, characterized by the highest *δ*_532_, also supports this finding. Moreover, the lidar ratios at both 355 and 532 nm also have a minima in the center of the layer because the lidar ratio of dust is lower than that of smoke. The contributions of dust and smoke particles to the aerosol backscattering and extinction coefficient at 532 nm evaluated from particle depolarization ratio agree with the values provided by the model. Of course an analysis of only one episode is not sufficient for broad conclusions regarding how well the model reproduces the vertical distribution of particle properties. More measurements at different locations are needed. However, the results presented here demonstrate that observations and the MERRA-2 model contribute in a complementary way, allowing the separation of the contributions of different chemical component of the aerosol mixture.

The motivation for this work is to show that the aerosol transport model has sufficient skill to serve as an additional constraint in inversion of 3*β* + 2*α* lidar observations and development of such constrained inversion is in progress. Assimilation of lidar measured parameters in the model is the subject of our future efforts.

## Figures and Tables

**Figure A1.**
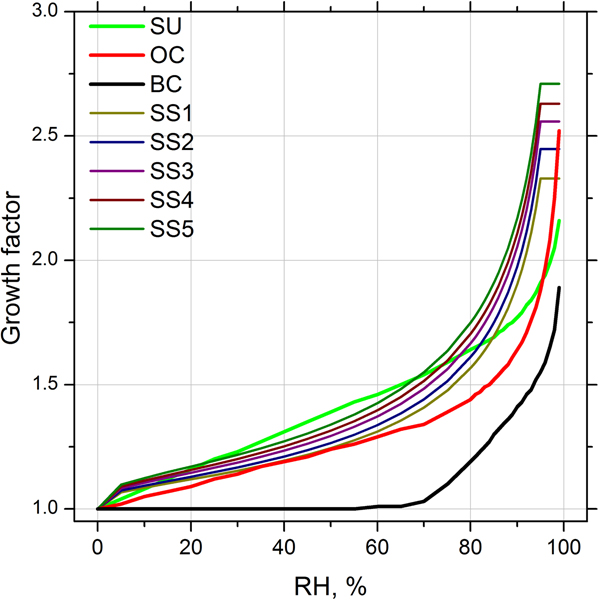
Dependence of the growth factor of organic carbon, black carbon, sulfates, and sea salt on relative humidity (RH) used in MERRA-2. For the sea salt the results are given for five size bins from Table A1. The growth factor increases with increase of bin number.

**Figure A2.**
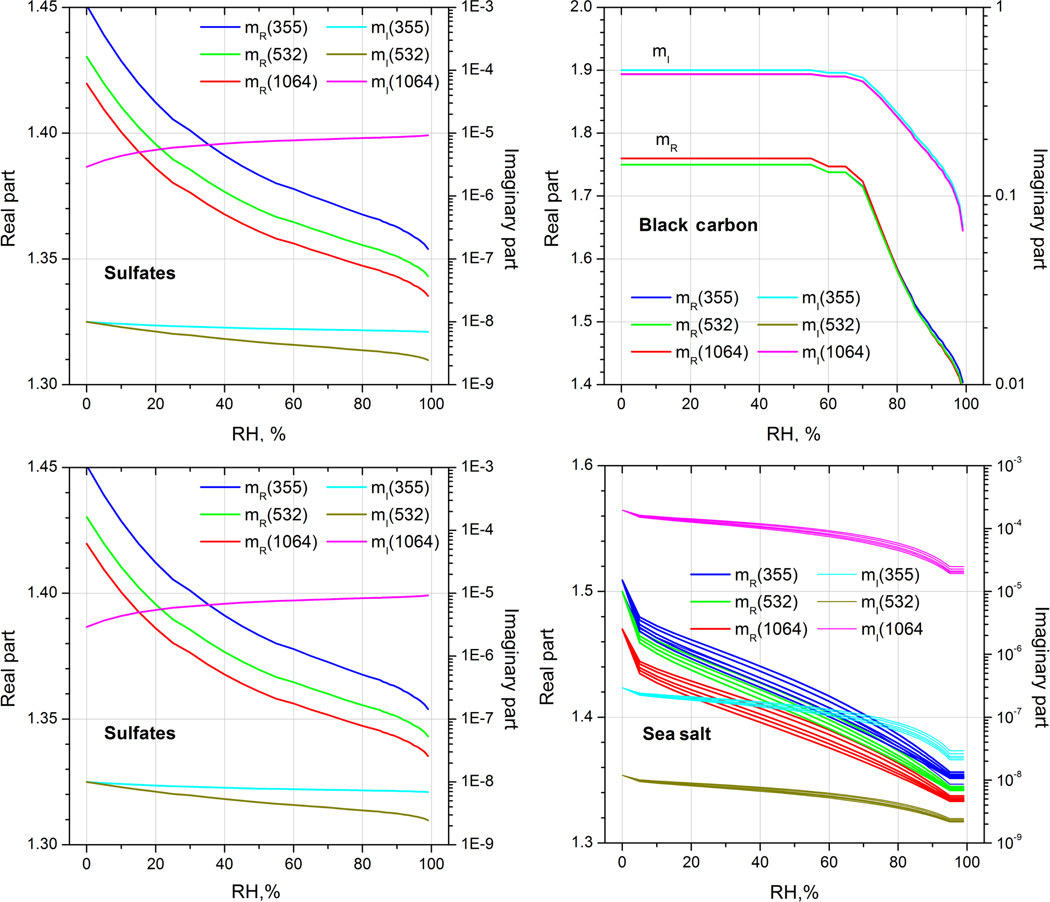
Dependence of the real and imaginary part of the refractive index of organic carbon (OC), black carbon (BC), sulfates (SU), and sea salt (SS) on relative humidity (RH) used in the MERRA-2 model. For the sea salt the results are given for five size bins from Table 1. Both *m*_R_ and *m*_I_ decrease with bin number increasing.

## Appendix A: Optical properties of aerosol components in MERRA-2 model

Table A1 summarizes the main characteristics of five aerosol components: dust, sea salt, black carbon, organic carbon, and sulfates used in MERRA-2 model. For dust and sea salt five size bins are considered. All values are given for the relative humidity of 0. Thus OC, BC, and SU with the effective radii of 0.09, 0.04, and 0.157 μm, respectively, are presented by the fine fraction only, while dust and sea salt contribute to both fine and coarse fractions.

The dust particles are assumed to be hydrophobic, but other aerosol components may present significant hygroscopic growth. To account for the effect of RH, the growth factor *g*, which is the ratio of particle radius at current RH to the dry particle radius, is introduced. Figure A1 shows dependence of the growth factor of different aerosol components on RH. For sea salt the results are given for five size bins from Table 1. Each bin has a different growth factor: *g* increases with increase of particle radius. Relative humidity modifies also the particle complex refractive index. Dependence of the real and the imaginary part of particle components on RH is shown in Fig. A2. For dry sea salt particles RI is supposed to be the same for all size bins. However, in the process of hygroscopic growth the RI of different bins behaves differently: both *m*_R_ and *m*_I_ decrease with bin number (radius) increasing.

**Table A1.** Parameters of the aerosol components, such as minimal radius (*r*_min_), maximal radius (*r*_max_), effective radius (*r*_eff_), and real (*m*_R_) and imaginary (*m*_I_) part of the refractive index at 355, 532, and 1064 nm used in MERRA-2 model. For dust and sea salt, five size bins are considered. All values are given for RH = 0.

Component		*r*_min_ μm	*r*_max_ μm	*r*_eff_ μm	*m*_R355_	*m*_R532_	*m*_R1064_	*m*_I355_	*m*_I532_	*m*_I1064_
Dust	Bin 1	0.1	1.0	0.64	1.53	1.53	1.53	0.007	0.0026	0.0022
	Bin 2	1	1.5	1.32	1.53	1.53	1.53	0.007	0.0026	0.0022
	Bin 3	1.5	3.0	2.30	1.53	1.53	1.53	0.007	0.0026	0.0022
	Bin 4	3.0	7.0	4.17	1.53	1.53	1.53	0.007	0.0026	0.0022
	Bin 5	7.0	10.0	7.67	1.53	1.53	1.53	0.007	0.0026	0.0022

Sea salt	Bin 1	0.03	0.1	0.08	1.51	1.50	1.47	2.9E-7	1.2E-8	1.97E-4
	Bin 2	0.1	0.5	0.27	1.51	1.50	1.47	2.9E-7	1.2E-8	1.97E-4
	Bin 3	0.5	1.5	1.07	1.51	1.50	1.47	2.9E-7	1.2E-8	1.97E-4
	Bin 4	1.5	5	2.55	1.51	1.50	1.47	2.9E-7	1.2E-8	1.97E-4
	Bin 5	5	10	7.3	1.51	1.50	1.47	2.9E-7	1.2E-8	1.97E-4

OC		0.01	0.29	0.09	1.53	1.53	1.52	0.048	0.009	0.016
BC		0.01	0.29	0.04	1.75	1.75	1.75	0.46	0.44	0.44
SU		0.01	0.29	0.157	1.45	1.43	1.42	1E-8	1E-8	2.9E-6
